# Clinicians’ decision-making about broad-spectrum antibiotic prescribing for suspected maternal sepsis during childbirth in the UK: a qualitative study

**DOI:** 10.1136/bmjopen-2025-110559

**Published:** 2026-07-17

**Authors:** Carol Kingdon, Benjamin Greenfield, Caroline Cunningham, David Freeman, Damien Ming, David Lissauer, Alison Holmes, Abi Merriel

**Affiliations:** 1 Department of Women's & Children's Health, Institute of Life Course & Medical Sciences, Faculty of Health and Life Sciences, University of Liverpool, Liverpool, UK; 2 Liverpool Women’s University Hospital NHS Foundation Trust, University Hospitals of Liverpool Group, Liverpool, UK; 3 Centre for Antimicrobial Optimisation, Imperial College London, London, UK; 4 National Institute of Health Research (NIHR) Applied Research Collaboration (ARC) North West Coast, Liverpool, UK; 5 The David Price Evans Global Health and Infectious Diseases Unit, Faculty of Health and Life Sciences, University of Liverpool, Liverpool, UK; 6 National Institute of Health Research, Health Protection Research Unit in Healthcare Associated Infection and Antimicrobial Resistance, London, UK

**Keywords:** OBSTETRICS, Antibiotics, Health Services, QUALITATIVE RESEARCH

## Abstract

**Abstract:**

**Objective:**

Sepsis management and antimicrobial resistance (AMR) are linked priorities. Early identification and treatment of sepsis with broad-spectrum antibiotics are key to reducing morbidity and mortality. We aimed to investigate clinicians’ decision-making about broad-spectrum antibiotics for suspected maternal sepsis in women admitted to hospital for childbirth, to identify if, how and why overtreatment may occur.

**Design:**

Qualitative study. Semistructured interviews were conducted online, transcribed verbatim, coded in MAXQDA and analysed using the thematic Framework Approach.

**Setting:**

National Health Service (NHS) in England, Scotland and Wales, March–June 2024.

**Participants:**

24 clinicians, purposively sampled for representation from relevant professions with roles in decision-making (n=15 obstetricians; n=4 anaesthetists; n=3 midwives; n=2 microbiologists).

**Results:**

Participants report increasing numbers of women in labour are being prescribed broad-spectrum antibiotics for suspected maternal sepsis per guidelines. Fear of missing sepsis, absence of evidence about sepsis during labour and beliefs about antibiotic use informed clinicians’ views, impacting workloads and women’s experiences. 9/24 participants said overtreatment is a problem; 21/24 believed overtreatment occurs. Clinicians’ beliefs, views and experiences are reported in three themes that provide a framework to explain how clinicians navigate guidelines including; why overtreatment can occur (Theme 1: The sepsis guideline is more than a guideline); how decisions to prescribe broad-spectrum antibiotics during labour are made (Theme 2: Trying to balance the whole picture) and where clinicians’ think the bigger picture problems lie (Theme 3: If we continue down this path where might we be). The framework encapsulates clinicians’ tacit knowledge governing decision-making and guideline use.

**Conclusions:**

Clinicians link priorities for sepsis management and AMR, but antimicrobial stewardship comes second to concerns about missing sepsis in high-stakes maternity care. Overtreatment may be inevitable until better evidence to support decision-making for suspected maternal sepsis and point-of-care diagnostic tests for women in labour exist and are embedded in clinical guidelines and clinicians’ mindlines.

**STRENGTHS AND LIMITATIONS OF THIS STUDY**

- A key strength of this study is its interdisciplinary approach, combining medical and social sciences, to investigate the linked health priorities of preventing morbidity and deaths from sepsis and addressing antimicrobial resistance.
- A further strength of the study is that its qualitative design illuminates how maternity clinicians navigate intrapartum guidelines in the context of uncertain evidence, demonstrating the depth of reasons for prescribing broad-spectrum antibiotics during labour.
- The study includes a national sample of obstetricians, midwives, microbiologists and obstetric anaesthetists with representation across regions, but it is a limitation that only three midwives participated.

## Introduction

 Maternal sepsis, life-threatening organ dysfunction resulting from infection during pregnancy, childbirth, postabortion or postpartum, is a leading global cause of maternal morbidity and mortality, causing 17 000 deaths annually.^1^,^3^ It remains a persistent leading direct cause of maternal death in the UK, with 25 sepsis-related maternal deaths in 2020–2022.^4^ Most maternal sepsis occurs intrapartum or post partum.^5^ Women in labour are more vulnerable to severe infection because of their relative immunosuppression, physiological labour processes and exposure to medical intervention.^6^ In the UK, risk factors for severe sepsis include ethnicity and deprivation, with black and other ethnic minority groups conferring a twofold increased risk.^7^,^9^ Neonatal complications include respiratory distress, hypoglycaemia, cerebral palsy, neurodevelopmental delay and death.^1^ Surviving sepsis campaigns stress prompt diagnosis and broad-spectrum antibiotic administration.^10 11^ However antimicrobial resistance (AMR) is an increasing problem and could become the leading cause of death worldwide by 2050.^12^ In 2019, for every sepsis death, the probability that the organism causing the infection was drug-resistant was 25% higher compared with 1990.^13^ With few new antimicrobials in development, AMR and sepsis are now linked national and global priorities.^14^,^16^

Overdiagnosis and overtreatment are widely acknowledged to be complex problems that are not easy to define. Overdiagnosis and the overtreatment that follows refer to situations where a diagnosis is technically correct under current clinical standards yet is unlikely to benefit the patient. In such cases, the condition identified may never have caused symptoms or harm, and the interventions that follow can expose the patient to unnecessary risks, side effects or psychological burden.^17^ There is evidence of overtreatment of suspected sepsis using broad-spectrum antibiotics across medical specialities where clinicians often make antibiotic decisions while facing diagnostic uncertainty.^16 18^ General adult inpatient studies report that up to 50% of patients treated with broad-spectrum antibiotics for sepsis do not have sepsis.^16^ Up to 70% of intensive care unit (ICU) patients receive antimicrobial therapy, despite up to half never having infection confirmed.^18^ In maternity, a 2020 Scottish cohort study reported a sepsis incidence of 331 per 10 000 pregnancies,^19^ far higher than previous studies reporting 10 per 10 000 pregnancies.^19 20^ The study included all suspected or confirmed cases treated with antibiotics. In our recent study of 22 662 births, 36 of 839 women treated for suspected sepsis during labour had pathogenic positive blood cultures.^21^ During labour, physiological changes can make commonly used biomarkers difficult to interpret. National guidelines in the UK include modifications for pregnant women but rely on observations from non-pregnant populations.^22^,^25^ As an example, National Institute for Health and Care Excellence (NICE) recommend Lactate ≥2.0 mmol/L as a diagnostic marker. However, during the exertion of labour and birth, it is likely that a mother’s lactate will rise.^26 27^ The 2024 Royal College of Obstetricians and Gynaecologists guideline recognises this and mentions ≥4.0 mmol/L.^24^ In 2024, NICE also revised its guideline, updating its recommendations for stratifying risk to optimise appropriate antibiotic administration and address overuse, but recommendations remain unchanged for pregnant populations because of insufficient evidence.^28^

The drivers of AMR include humans, animals and agriculture.^13^ In healthcare, antimicrobial stewardship is vital^29^ to reduce costs, AMR and adverse events.^30^ However, changing hospital antibiotic use is complex, influenced by cultural, organisational and individual factors.^31^ The UK’s national action plan for AMR 2024–2029 includes targets to increase healthcare professionals’ knowledge of AMR and enhance decision support tools for optimal antimicrobial use.^32 33^ In-depth knowledge from within specialities may translate into more workable interventions for decision support. Little is known about how maternity care clinicians balance timely suspected sepsis treatment with appropriate broad-spectrum antibiotic use during labour.^34 35^ This study aimed to explore views and decision-making experiences of doctors and midwives about broad-spectrum antibiotic prescribing for suspected maternal sepsis in women admitted to hospital for childbirth, to identify if, how and why overtreatment may occur.

## Methods

This study is part of a programme of research seeking to advance the management of maternal, fetal and neonatal infection.^34^ The exploratory design was intended to inform implementation considerations for point-of-care interventions to support decision-making.

### Study design

This qualitative study used semistructured interviews and is reported using the Standards for Reporting of Qualitative Research (online supplemental table 1).^35^

We applied the concept of mindlines, meaning collectively reinforced, internalised, tacit guidelines used to support complex clinical decision-making.^36^,^39^ The mindlines paradigm originates from an ethnographic study of general practice which showed how clinicians relied less on research findings, clinical guidelines and other types of formal knowledge, and more on brief reading, interactions with each other, opinion leaders and patients; sources of largely tacit knowledge built on their early training, their own and their colleagues’ experiences.^37^ Mindlines enable understanding of the social construction of knowledge and the process of evidence interpretation.^38^,^42^ In other words, knowledge-in-practice-in-context.^36^

### Reflexivity

When conducting qualitative research, teams should engage in reflexivity to account for how subjectivity shapes their inquiry.^43 44^ We are an interdisciplinary team from sociology (CK), obstetrics (AM, BG and DL), infectious diseases (DM and AH), midwifery (CC) and electrochemistry (DF). Our preexisting beliefs encompassed clinical concerns about overtreatment (AM and BG), gaps in knowledge about physiological labour and sepsis (AM, BG, DL, DM and CK), persistent inequalities in maternal deaths from sepsis (CK, AM, CC and DL) and scope for new point-of-care diagnostic technologies during labour to address early sepsis management and AMR (AH, DF, DM, AM, BG and CK).^34^ We remained aware of these prior beliefs and took steps to mitigate professional biases. All interviews were conducted by the non-clinician in the team and we chose a rigorous, stepwise approach to data analysis to ensure that we were not over-interpreting data that supported prior views or overlooking data that ran contrary to our beliefs.

### Sample and setting

This research involved clinicians working in NHS hospitals providing care for pregnant women during labour. We sought participants from professions involved in the broad-spectrum antibiotic prescription decision-making process. In the UK, this includes senior input from consultant obstetricians and anaesthetists (10+ years’ experience), aided by microbiologists (usually also at consultant level); obstetric and anaesthetic early-grade (<4 years’ experience) and middle-grade (5–7 years’ experience) trainees; and midwives of all grades who will request medical review for suspected sepsis. Several recent systematic reviews have examined sample size in qualitative research, which we drew on when writing our protocol. Per protocol (online supplemental file 2), funding award and reviews of optimum sample size for qualitative interview research, we sought a maximum sample size of 25 interview participants.^45^,^48^

**Figure 1:**
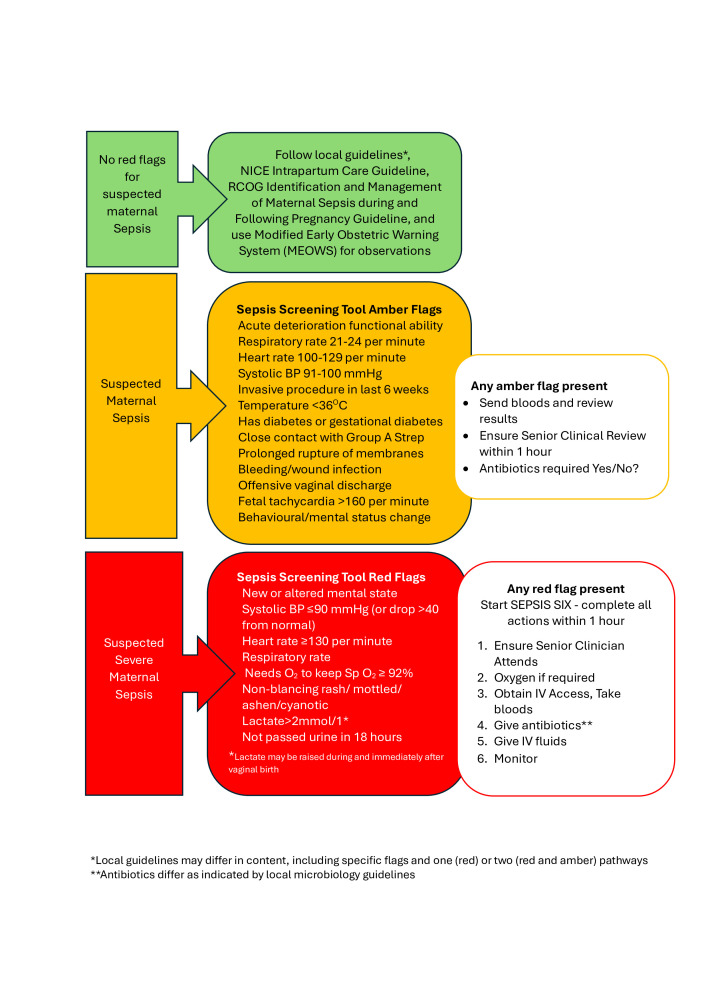
Management of suspected maternal sepsis. *Local guidelines may differ in content, including specific flags and one (red) or two (red and amber) pathways. **Antibiotics differ as indicated by local microbiology guidelines. BP, blood pressure; IV, intravenous; MEOWS, Modified Early Obstetric Warning System; NICE, National Institute for Health and Care Excellence; RCOG, Royal College of Obstetricians and Gynaecologists; SPo2, Peripheral capillary oxygen saturation.

Figure 1: Management of suspected maternal sepsis shows the pathways for intrapartum care, identification and management of maternal sepsis.

Study information was shared via national clinical networks and participants self-identified to the study team. A snowball sample ensued from which a purposeful sampling matrix was used to ensure representation from England, Scotland and Wales, hospitals with <3000, 3000–6000, >6000 births per annum, different grades and length of NHS service.^45^,^48^ The purposive sampling frame was principally designed to ensure breadth and depth of representation among obstetricians because they are the main prescribers of broad-spectrum antibiotics during labour in the UK. The inclusion of anaesthetists, midwives and microbiologists applied the same principles of geographical spread.

### Data collection

CK conducted all semistructured interviews online after gaining informed consent. Participants provided verbal consent via an electronically recorded and documented process. The interview guide (online supplemental file 3) was informed by medical and social science literature concerning sepsis,^49^ antimicrobial stewardship, AMR and mindlines.^39^ It included prompts about the knowledge clinicians draw on in their decision-making about when to prescribe, when not to, and when to step down. Interviews lasted between 25 and 55 min and were undertaken in 2024.

### Data analysis

All interviews were recorded and transcribed using Microsoft Teams and data was uploaded into MAXQDA for thematic qualitative data analysis using the Framework Approach.^50 51^ Data collection and familiarisation were simultaneous for the first 10 interviews from which a thematic framework was constructed to encapsulate a priori issues (informed by the questions asked), emergent issues (raised by participants) and analytical themes (arising from recurrence or patterning in data). Interviews continued until no new insights emerged (data saturation).^46 48^ The final framework matrix was agreed by two authors (CK and AM) before the indexing of all transcripts (codes in MAXQDA Analytics Pro 24.2.0). CK systematically charted data, from which mapping and interpretation generated final themes. All authors reviewed the final themes.

## Results

A total of 24 clinicians from all relevant professional groups, from hospitals across England, Scotland and Wales, participated in this study. Most clinicians worked in units with >6000 births a year; length of NHS service ranged from 0 to 9 to 30+ years (table 1).

**Table 1:** Participant characteristics

Profession	Total				
		Obstetrics	Midwifery	Anaesthesia	Microbiology
Participants	24	15	3	4	2
Sex					
Male	7	3	0	3	1
Female	17	12	3	1	1
Grade					
Consultant	14	8		3	2
Trainee (ST2–ST6)	7	7		1	
Band 7	1		1		
Band 6	2		2		
Length of NHS service					
0–9 years	8				
10–19 years	9				
20–29 years	5				
30+ years	2				
Size of unit					
≥6000	15				
≤3000–6000	7				
≤3000	2				
Region					
North of England	8				
South of England	9				
Midlands	4				
Scotland	2				
Wales	1				

NHS, National Health Service.

Our overarching finding was that clinicians feel compelled to start broad-spectrum antibiotics, governed by guidelines due to a fear of the consequences of missing sepsis, coupled with a high index of suspicion in pregnant populations, alongside a lack of evidence about normal physiological labour progress and pathological changes associated with maternal sepsis. The likelihood of adverse reactions and AMR was perceived as less risky than missing sepsis.

Participants perceive that increasing numbers of pregnant women in labour are being prescribed broad-spectrum antibiotics for suspected maternal sepsis. However, *“There’s no actual clear evidence that this is what we should be doing in maternity. It’s just something that we’ve kind of extrapolated from a very different setting” (ID05*). It is not the strength of evidence underpinning guidelines that makes current maternal sepsis management the right care, for the right patient, at the right time,^9 13^ but the absence of evidence to distinguish physiological labour from suspected sepsis. Diagnostic uncertainty and high stakes mean clinicians need guidelines to defend actions and direct decision-making.

Nine participants explicitly said that they thought over-prescribing leading to overtreatment occurs in maternity and perceived this as a problem (IDs 02, 03, 04, 05, 16, 18, 20, 22, 24). Direct quotes aligned to this include *“100% I think we overprescribe in maternity”* (ID05) and *“we are probably over treating quite a lot of people”* (ID20). 12 participants (IDs 06, 07, 09, 10, 11, 12, 13, 14, 15, 17, 19, 21) were sceptical about current use, but their views were more aligned to this quote “*because the risk of not doing it is so high that the benefit-risk equation always favours giving it” (ID09*). The views of the other three participants (IDs 01, 08, 23) were more guarded, reporting they did not recall *“it was inappropriate seeing someone that had been started on broad-spectrum antibiotics” (ID01*).

Anaesthetists had a broader view of sepsis management than midwives and obstetricians. Two anaesthetists involved in cross-speciality working, including ICU shifts, did not view overtreatment as a problem, whereas two consultant anaesthetists working exclusively in maternity do. The views of the two consultant microbiologists and midwives were similarly split. Four consultant obstetricians and one trainee thought that overtreatment occurs and is a problem. The quotes below show the range and strength of feelings about potential overtreatment:

*Now sometimes you can have a labour ward, you can have 10 women in labour and they’re all on antibiotics. My personal opinion is that we over-treat intrapartum infection and that we are making normal women really abnormal as a result of the criteria for giving antibiotics. And I think that there is a significant harm from that over-treatment. So I think I would like to see us doing that less. (Obstetrician, ID04)*

*I don’t get the impression it’s a big problem. (Microbiologist, ID23)*

*Guidelines are there to protect. And so sometimes you just have to take the hit on the fact there is a little bit of overprescribing because the balance between that and [ ] missing things is you know why the safety feature is there. (Obstetrician, ID17)*

Many participants acknowledged a solution to overtreatment is, as this participant said “*an intervention, bedside test or quick lab result that was binary, sepsis, not sepsis” (ID20*).

**Figure 2:**
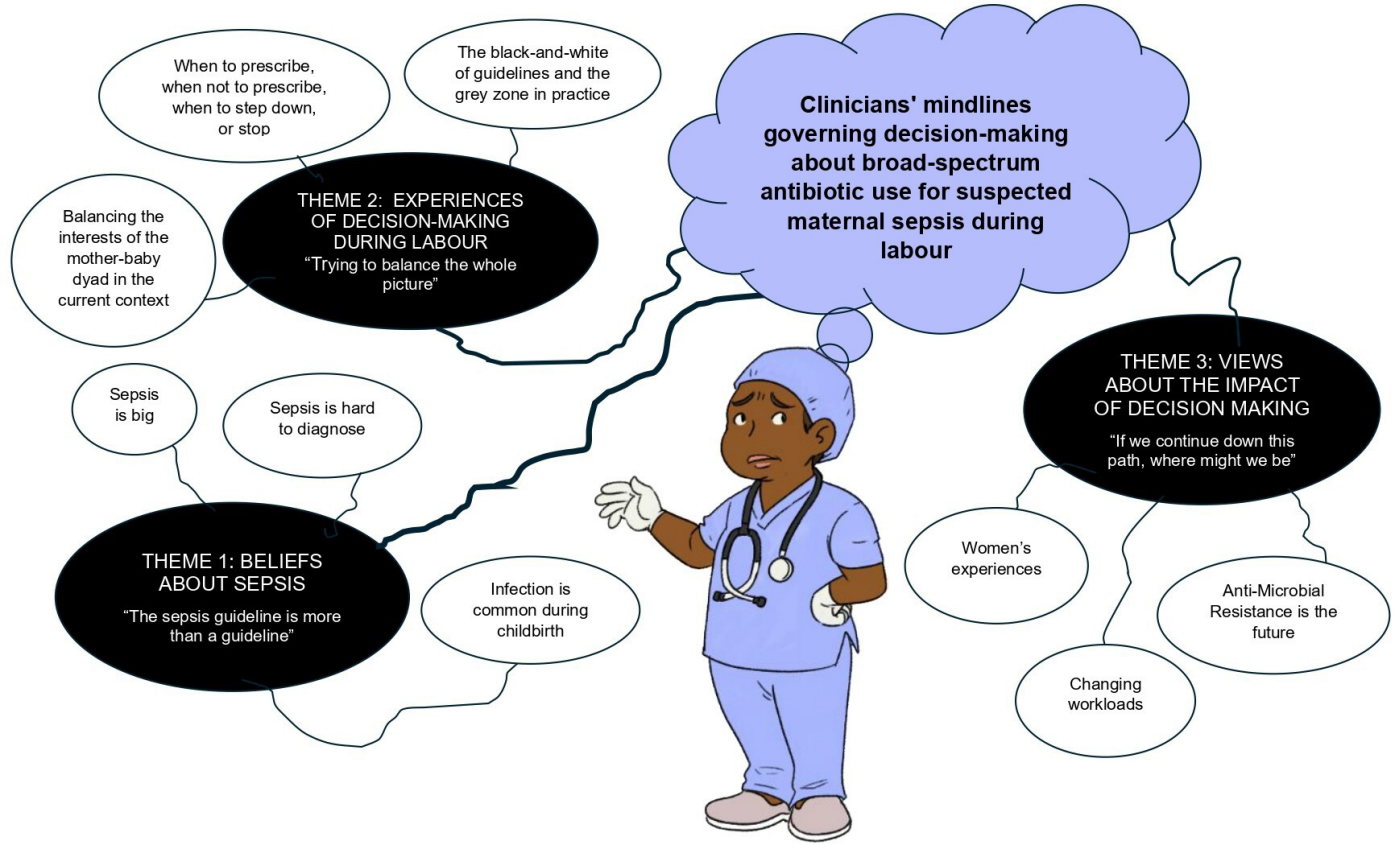
Thematic framework map of themes and subthemes.

Figure 2: Thematic framework map of themes and subthemes shows the thematic framework depicting clinicians’ mindlines that governed decision-making.

### Theme 1: clinicians’ beliefs about sepsis: ‘*the sepsis guideline is more than a guideline’*

This theme encapsulates why clinicians use the sepsis guideline as a mandate, and why overtreatment may occur. Although some participants stated that a “*guideline should just be a guide,”* this ran contrary to what all participants said about why sepsis guidelines are adhered to.

*There are clinical decisions that my seniority will trump, for example. But the sepsis [guideline] that’s not the case, not in my experience… You just have to give it. You don’t have to think it; it’s not really a decision… (Obstetrician, ID09)*

*The risk of harm vs the risk of benefit of antibiotics is perceived to be quite low. I think there’s fear associated with missing sepsis. There’s been such a drive about sepsis… (Obstetrician, ID02)*

An overview of subthemes, codes and illustrative quotes can be found in table 2.

**Table 2:** Clinicians’ beliefs about sepsis and why sepsis guidelines are more than guidance

Subtheme	Codes	Supporting quotes
1.1 Sepsis is big	Power of word, public campaigns, medical training and the NHS	1. “There’s been such a drive about sepsis in the general public.” (ID02)
		2. “You hear a lot of people, whenever you say the word sepsis to patients, automatically are terrified of that word in itself. There’s a lot of fear associated with it because everybody’s heard a horror story or knows somebody in the public who’s had sepsis. So I think there is that aspect of it.(ID15).
		3. “The wording around sepsis is funny. You say the word sepsis to a patient; they freak out a little bit.” (ID12)
		4. “Because someone says the word sepsis and then we’re committed to that SEPSIS-SIX bundle.” (ID05)
		5. “We are taught about sepsis at medical school and in our training, whatever profession or speciality… there are campaigns “Surviving Sepsis”, “The SEPSIS-SIX” (ID01)
		6. “The whole sepsis thing. SEPSIS-SIX has become very prominent. At the forefront of everybody’s minds.” (ID16)
		7. “It’s on the side of all the ambulances, has been for a number of years.” (ID08)
	Fear of missing sepsis and a fatal outcome	8. “It’s getting the antibiotics in a timely manner, isn’t it to prevent those deaths.” (ID07)
		9. “We’re talking about, you know, we’re talking about death, rather than days off work.” (ID16)
		10. “We know that there is significant harm from maternal deaths.” (ID19)
		11. “Sepsis was, well probably sepsis has always been, one of the big, most common causes of maternal death.” (ID18)
		12. “My kind of early years of training followed on the back of sepsis being leading direct cause of death.” (ID05)
		13. “I think there’s fear associated with missing sepsis.” (ID02).
		14. “I think that a big fear is you’re going to miss it.” (ID17)
	High Index of suspicion, actual incidence unclear	15. “I think we’re more vigilant, aren’t we.” (ID24)
		16. “Probably use it [Sepsis Six])2 or 3 times a day at least, but that’s just in a day, there’s the nighttime as well. Probably about four or five times for 24 hours on our site.[ ] There are quite a lot of people that we give antibiotics to, who then get better in the following 24 hours, and you don’t know whether that’s because of the antibiotics or not, or whether they would have got better anyway.” (ID03)
		17. “I don’t know the data for the reduction in sepsis deaths across all of medicine since these things have been in place, but I assume there is one. Yeah.” (ID01)
		18. “Far more women are diagnosed with infection or classified as potentially septic than are not than 15–20 years ago. Whether that’s had a significant impact on mortalities? I mean M-BRRACE would suggest not. [ ] I wonder how many women we give antibiotics to that we didn’t need to and how many women we should have given antibiotics to that we didn’t. And how would that tally up with neonatal outcome in turn.” (ID16)
		19. “The thing that you would really want to know is, if you didn’t treat any of those patients, how many of them would go on to have severe sepsis? You’re trying to prevent something more serious developing. I certainly think that on delivery suite we over-prescribe the red sepsis antibiotics. Whether that means that those patients don’t need antibiotics at all or not is a tricky question. But do we need to hit everyone with, you know, triple antibiotics, sort of the Domestos level that we do? I think that’s probably more of the problem.” (ID06)
1.2 Sepsis is hard to diagnose and suspected sepsis easy to treat	Sepsis as mimic	20. “It can be more difficult sometimes to diagnose sepsis [in pregnant patients] than in non-pregnant patients because of some of the overlap with normal physiological changes in physiology are the signs that we associate with sepsis.” (ID01)
		21. “It’s very difficult in pregnant women because they can deteriorate quite quickly. I suppose if you ask the question about the non-obstetric population, there is a good on-going history with clinical evidence that there is an infection prior to administering antibiotics.” (ID08)
		22. “You know, it is a great mimic. I’ve missed sepsis in other contexts [ ] you know, there’s always a confounder, and pregnancy is a great confounder” (ID20)
		23. “In COVID times, patients present with tachycardia, temperature, cough and you might start the antibiotics, but once you get the viral swabs back, you would stop them at that point.” (ID06)
		24. “We don’t truly know the accuracy of the diagnostic algorithm that we use to treat people.” (ID10)
	Time pressure to diagnose	25. “As with non-pregnant patients the standard is we should do this within an hour of diagnosis. I don’t know where it’s from, but I know the first hour of suspicion of sepsis we should administer the antibiotics.” (ID01)
		26. “We are trained that there’s that golden hour where you’re to give IV antibiotics if you think sepsis.” (ID12)
		27. “We’ve got that pressure of the golden hour, as it were, the targets that we are expected to meet.” (ID16)
		28. “Taught you must get the antibiotics within the first hour. That’s the golden hour. It’s The SEPSIS-SIX.” (ID17)
		29. “The thing in labour is you haven’t got that long to wait and see because you know it’s a finite period of time. [ ] It’s the pressure of time. Like everything is easy to just quickly get onto it. F1, F2, your main job in life is doing bloods, prescribing, doing the more simple things. You’re not necessarily thinking.That’s what made me start thinking about this, because as an SHO I was like, oh, here I go for another sepsis screen. Why are we doing this? I think everyone would say that they feel like we have to prescribe, though.” (ID07)
	Period of lag on definitive diagnosis	30. “The nature of microorganisms and bacteria is generally we need 24 hours to be able to grow them and to know what the cause is. So we’re always working in a period of lag before we know exactly what we’re dealing with, so we tend to go for this sort of slightly safety net approach. The downside is that lots of ladies get exposed to broad-spectrum antimicrobials which they possibly don’t need.” (ID18)
		31. “I don’t know how good we are actually going back and truly retrospectively looking at certainly in our own patients who actually had anything cultured, anywhere.” (ID16)
		32. “I don’t know the numbers, but the majority if you looked up someone’s blood cultures. The majority don’t have anything in their cultures. And the same with babies.” (ID22)
1.3 Infection is common	Pregnant women are at higher risk of infection	33. “As a pregnant woman you are immune regulated, your immune system’s dampened down because you’re growing something that’s half you and some half somebody else’s DNA so you’re just so much more likely to get any kind of infection.” (ID02)
		34. “I think there’s a lot more women with risk factors now. We’ve got high induction rates. We’ve got delays of people, having multiple vaginal examinations, rupturing the membranes, but then not being able to get in for an induction for you know, however long.” (ID21)
		35. “The risk factors would be a lady that’s PROM [Premature Rupture of Membranes]. Always a prolonged labour… We’ve had some very sick women with that [birthing outside of guidance]” (ID24)
	Iatrogenic sepsis	36. “I think the problems start way before you’re at the point of prescribing the antibiotic. I think you know, unnecessary inductions and causing unnecessary vaginal examinations, which is causing unnecessary artificial rupture of membranes and all these things.” (ID22)
		37. “So an awful lot of ladies get induced now, (…) every time we do an examination, you’re increasing the risk of infection.” (ID24)
		38. “How many would have been fine without intervention? Like I think a lot of things are iatrogenic, because we’re monitoring obs[ervations], because they’re in a unit, they could be factors that we have done interventions.” (ID13)
	Conflating infection with suspected sepsis and severe sepsis makes antibiotics an easy intervention to give	39. “To me, what my idea of sepsis is, is someone very sick, very ill, where infection is spread through the whole body, not localised (…) That’s why I feel like we shouldn’t call it a sepsis pathway, an infection pathway would be better.” (ID03)
		40. “People think that they’ve had sepsis and labour when they’ve not, they've had a suspected infection and we've treated them for that. (…) we've lost the way a little bit and in amongst that I think we have, we have, ended up overprescribing quite a lot.” (ID05)
		41. “I’ve never really seen the word infection written. I feel like a lot of the times we’ve said, suspected, intrapartum sepsis, most of the women that we give that label of sepsis to, do not meet that criteria.” (ID13)
		42. “It’s a drip, it’s another bag of fluid going in your arm. It’s not like a [caesarean] section or a forceps or something. I don’t think it’s particularly perceived as an intrusive or particularly negative intervention.” (ID09)
		43. “It feels like an easy intervention to recommend and instigate [antibiotics] in comparison to some of the other things we do.” (ID02)
		44. “The minute they have a temperature [we] say they have sepsis (…) And actually it’s just an infection. It’s really important. You know they do have an infection, but it seems that anything that triggers anything, it’s called sepsis. It’s replaced the word infection.” (ID17)
		45. “We’re really keen in our unit, making sure that we use the terminology correctly. So pyrexia in labour, potential infection vs sepsis. You know we you only get a handful of people who have sepsis with the end organ dysfunction by definition. Handful of people a year who end up on ITU needing intubation and support.” (ID10)

NHS, National Health Service.

#### Subtheme 1.1: sepsis is big

All participants described the power of campaigns, which have raised public awareness of sepsis; ‘Think Sepsis’ is now entrenched in hospital culture and guidelines; and ‘SEPSIS-SIX’ is embedded into medical school curriculum and hospital mandatory training. Clinicians perceive public awareness of sepsis is high, influenced by high-profile cases in the media, ground-floor visibility on the side of ambulances and in hospital wards (table 2, quotes 1–7). While only a few participants had witnessed deaths or knew colleagues who had, all participants recognised sepsis as a leading cause of maternal death in the UK. Clinicians feared missing sepsis, irrespective of profession, specialty, grade or organisation (table 2, quotes 8–14). Yet only two participants knew the actual incidence of maternal sepsis in their organisation. Many participants (n=15) worked in large units (>6000 births a year) where they reported between 2 to 3 women would be started on the red sepsis pathway in a 12-hour shift. The difficulty recognised by many clinicians was not knowing how many cases of sepsis are averted by early intervention or how many slip through the net (table 2, quotes 1–19).

#### Subtheme 1.2: *all sepsis is hard to diagnose*

Anaesthetists were most vocal about how difficult it is to diagnose sepsis in any patient, and how it can be even more difficult in pregnant patients, because normal physiological changes during pregnancy are the same signs that general medicine associates with sepsis. Reference to The SEPSIS-SIX was ubiquitous, although many questioned its diagnostic value during labour (table 2, quotes 20–24). 17 clinicians identified as having ‘grown up’ in the culture of the SEPSIS-SIX that should be completed within 1 hour. 14 clinicians spontaneously used the phrase ‘the golden hour’, which was described as universally good, a time pressure that forces decision-making, an NHS-system-wide target, and a quality improvement priority (table 2, quotes 25–29). Following guidelines provided a safety net via algorithms to force timely decision-making, diagnosis and treatment. As one microbiologist said, blood cultures are ‘the gold standard of proving somebody has sepsis’ they can identify the causative bacterial organism that determines which antibiotic to use to treat. However, while all clinicians described taking cultures and sending them to the microbiology laboratory per guidelines and the SEPSIS-SIX, cultures take 12–24+ hours to grow. Participants reported they do not have the time afforded to other specialties, they must make urgent decisions, for example deciding whether to expedite birth, rather than being able to observe the patient. In this complex scenario broad-spectrum antibiotics are the ‘just-in-case, catch-all’ option (table 2, quotes 30–32).

#### Subtheme 1.3: *infection is common during childbirth*

All clinicians believed pregnant women are high-risk for sepsis, some said increased interventions during labour are causing more sepsis, and many suggested sepsis guidelines are conflating infection and suspected sepsis with severe sepsis. Obstetricians, anaesthetists and microbiologists emphasised the seriousness of pregnant women being immunocompromised and more susceptible to infection and rapid sepsis onset (table 2, quotes 33–35). They also highlighted the intersections between general health (diabetes, body mass index), pregnancy health (including infections before labour) and labour complications (premature rupture of membranes, instrumental birth) that increase sepsis risk. One microbiologist and one obstetrician believed sepsis incidence and AMR in pregnant populations are rising because of global migration and health tourism. Five clinicians (obstetricians n=2, midwives n=3) believed ‘iatrogenic sepsis’ can occur(table 2, quotes 36–38). Many clinicians felt that current management is inflating perceptions of sepsis prevalence and stressed the importance of language in shifting beliefs about sepsis suggesting we should not call it a sepsis pathway; an infection pathway would be better (table 2, quotes 39–45). Clinicians believed there are important differences between talking about ‘true sepsis’, suspected sepsis and infection. Senior obstetricians and midwives worried slippage in these distinctions desensitises clinicians to the gravity of sepsis and encourages a lower threshold for intervention and treating all infections with broad-spectrum antibiotics. Clinicians recognised where they work can influence how they use these words, suggesting variation between hospitals and within clinical areas.

### Theme 2: clinicians experiences of decision-making during labour: *trying to balance the whole picture”*

This theme reports how decisions to prescribe broad-spectrum antibiotics during labour are made. When describing the care they provide during labour, clinicians drew on clinical acumen and previous experience, in tandem with guideline use. The experiences of clinicians caring for women during labour and the unique challenges it poses are encapsulated in the quotes below:

*You can score those patients and they can fit that red sepsis pathway… but really you need to take it in context with the clinical picture. (Obstetrician, ID19)*

*My feeling is that we over-treat people, but then the consequences of missing someone who has an infection that then becomes an infection that causes baby or maternal injury is also high, so there’s a balance and trade-off between over-treatment and under-treatment and working out where that line is, is the challenge. (Obstetrician, ID10)*

Three subthemes show how the balance favours following guidelines and prescribing broad-spectrum antibiotics for suspected maternal sepsis. See table 3 for subthemes, codes and illustrative quotes.

**Table 3:** Clinicians’ experiences of decision-making during labour and how clinicians balance the whole picture

Subtheme	Codes	Supporting quotes
2.1 The black-and-white of guidelines and the grey zone in practice	Guidelines as black and white	1. “There’s such a strict criteria in whether you commence on the sepsis pathway, it’s a lot more black and white than it was before.” (ID021)
		2. “I think there are guidelines for sepsis in most units that you’re working in, with quite strict criteria that you look through and you think does the patient score for this? Yes? Well then, there’s a chance it’s sepsis. We should give antibiotics.” (ID12)
		3. “What informs the decision-making process is the emphasis on sepsis, reading the numbers, sepsis pathways, and commencing the sepsis bundle. The triggers and parameters that are used, not don’t allow, but don’t necessarily encourage, clinical judgement. It becomes a bit of a tick box exercise. The default position is to commence broad-spectrum antibiotics.” (ID16)
		4. “I’m a robot when it comes to antibiotics on delivery suite and intrapartum, I follow the guideline, do what I’m told, we get them(broad-spectrum antibiotics)into patients when they tick a box according to the guideline.” (ID20)
	Nothing in labour is black and white	5. “You know, nothing is ever black and white in medicine, unfortunately (…) Are they [guidelines] written by people who really understand that pregnant women are different from non-pregnant women and that the changes, the lactates, are allowed to be normal rather than having to be pathology… I think it’s important to recognise the changes in the picture that you get because you want to pick up the ones who are at risk of developing sepsis, but you don’t necessarily want to start everybody on antibiotics… We need to know what’s normal in pregnancy. That’s what we need to know… and it’s difficult because no one ever likes to do stuff on pregnant women, do they? They never like to. You know, it’s very hard population to research, but we need some idea of where do we set our parameters really for what is normal.” (ID06)
		6. “In pregnant women it would be more permissible for women to have a slightly raised white cell count than it would for someone who’s not pregnant, because we expect that as part of the pregnancy profile. I think one of the problems is that we rely quite heavily on lactate. Lactate is a readily measurable bedside test, but it’s going to go up for lots of different reasons, but it’s a red sepsis flag.” (ID16)
		7. “As practitioners, you know we’re fairly simple as obstetricians we kind of like the RCOG or NICE telling us clearly what to do and I think the problem here is that there’s not really very kind of structured, clear clinical guidance as to what to do right, you know, suspected bacterial infection.[ ] It’s a tricky decision because we don’t have the perfect test…[ ] Labour’s quite a massive physiological challenge, but also it’s quite a variable one and it’s affecting different women differently depending on their reserve. A lot of the time they’re triggering because they’re also tachycardic, and it’s just a little bit self-fulfilling.” (ID05)
		8. “The one I disagree with colleagues on is this white cell in labour. What does that mean? Because I don’t think we know what the normal range is. And I think if the lactate is up, I would take that seriously. I don’t think there’s been enough research studies on what is a normal lactate in labour.” (ID09)
	Clinical acumen in the grey zone	9. “It’s just when they come it’s in the grey zone, the ones who’ve got a temperature of 37.9 or 38.1 or something, then it’s what are her other observations, not just the temperature. The observations that you’d normally diagnose sepsis in the normal adult population is different in pregnancy. Most of our MEOWS charts have changed now to incorporate that but where they got the two temperatures of 37.5 or one of 38 yeah, I don’t know.” (ID07)
		10. “But what we do know is that women that have epidurals, their temperature goes up… If you’ve had an epidural for 12 hours, there’s no question you’re going to be pyrexial. It’s just a known effect of epidural analgesia. So yeah that’s one thing I think yeah, I know why they’ve got a temperature of 38.1. We’ve had an epidural in for a while. That’s going to be the reason, but I think it’s still sensible to screen.” (ID08)
		11. “Temperature of a lady in the second stage, that’s quite a foolhardy thing to do really because their temperature always goes up in the second stage, like their lactate.” (ID22)
		12. “I occasionally do have consultants say it’s a one-off temperature we know that she’s obstructed, you don’t need to action that. But that happens in a minority of the time (…) I think it’s more of a seniority and a clinical acumen that if you can see that it’s just an isolated temperature in the context of what looks like an obstructed labour, slow progress, the decision to intervene to do a caesarean because you’ve got failure to progress”. (ID02)
		13. “I’ll identify my risk factors. I think, right. Well, she’s got PROM, you know, whatever. We’ll watch out and just alert the sort of junior midwives to do her temperature a little bit more often (…) A baby with a tachycardia and a mother with a tachycardia. I would really be quite concerned even if the temperature wasn’t up. Definitely. And especially with raised resps as well. (…) The first sign is foetal tachycardia, caught it loads of times like that. I’ve seen some very sick women.” (ID24)
2.2 When to prescribe, when not and when to step down or stop	Decision to prescribe	14. “Sometimes if the history is quite obvious, if their waters have gone for quite a long time and they’ve got high risk factors for infection, then more likely to. But then if they’re more likely to have a viral infection, I’d probably hold off. For instance, if they’ve got a bit of a sore throat, for instance. Sometimes I think maybe I’ve jumped on the gun and been quite quick to prescribe broad-spectrum antibiotics. And then the consultant might not have and then vice versa. But it’s difficult to know how things will evolve, from person to person.” (ID15).
		15. “If there’s temperature, changes in the blood, if the white cell count on the CRP is altered. If they’re having rigours, symptoms that point you to a particular source of infections so dysuria or productive cough, shortness of breath, abdominal pain, things like that, that the obstetricians are happy aren’t related to labour. Overall, I guess it’d just be the history and the symptoms put together with the sort of biochemical markers and then discussion with the obstetrician, sort of an MDT decision about whether to start antibiotics or not.” (ID06)
		16. “In the context of intrapartum sepsis for women who are labouring that would be guided by a multidisciplinary conversation and looking at the guidelines, with reference to knowledge of things that are contraindicated whilst there is still a foetus so for example, thinking co-amoxicillin and association with necrotizing enterocolitis for the neonate and understanding why that is the case in terms of how the guideline is written, for which antibiotics we would choose. So yes, mostly MDT discussions and guideline-based decision making. (ID20)
		17. “I think a lot of it is the things we’ve got in place, like the MOEWS chart and red sepsis pathway and everything. But ultimately I think a lot of it is your clinical judgement.” (ID22)
		18. “It’s actually quite difficult to not give antibiotics now where it never used to be. So before it was much more of an autonomous think about it decision. Whereas now to actually not give antibiotics is actually very difficult. You kind of have to give the antibiotics because that’s what the protocol says. Even if you don’t think that’s what the problem is.” (ID09)
	Decision not to prescribe	19. “I think that if we miss giving antibiotics as an option to women, then we could potentially allow a woman to get more sick before ultimately choosing to use broad-spectrum antibiotics and treating her then. I think the decision not to give antibiotics is often braver than the decision to give them.” (ID02)
		20. “I think when it comes to sepsis, it takes a really brave person to say we’ll just sit tight.” (ID21)
		21. “They look clinically well and their early warning score or MEOWS score is very low. I’m not suspicious from clinical examination or investigations that there is indeed a bacterial infection ongoing. If there is any clinical suspicion, I wouldn’t hesitate to give.” (ID08)
		22. “It’s really obvious that the heart rates going up and the blood pressures dropping because of the haemorrhage and because of this [sepsis].” (ID03)
	Decision to step-down or stop	23. “It does have to be that balance. I am I’m often uncritical of people who start with a broad spectrum in the context of sepsis, or on the way, you don’t know the cause. But I’m more critical of people who then don’t review that decision within 24–48 hours and say you know what the patients better, we don’t need to carry on that, or we may indeed have identified what the cause is and we can narrow it [which antibiotic] down. If we still don’t know the organism but the patient’s better, you’re out of that very risky situation.” (ID18)
		24. “I’m meeting this woman, the junior obstetrician, the junior anaesthetist overnight have started antibiotics, quite reasonably. Where I get to be more of an intelligent robot is in that stopping of the antibiotics and in those conversations around narrowing the focus of sources postpartum when they’ve got through the kind of the initial phase.” (ID20)
		25. “I think it’s easier the more senior you become to feel confident in making those decisions and saying, after someone’s had a couple of doses of IV antibiotics just to say, OK, we’ll just stop them. I’m not really worried about her, but do I think that an ST2 is going to do that if they’re going to review them in the ward? Probably not. Because they’re going to be more anxious about missing something or feeling like its quite a bold step just to stop antibiotics altogether after she’s had a couple of doses.” (ID05)
		26. “The obstetricians, on the basis that there’s no other clinical signs might say that’s chorioamnionitis because of the change in the clinical picture, so you might then take the patient to theatre, but actually at the emergency section there’s no evidence of chorio, so then you would stop and then repeat the blood tests (…) Everything could return to normal once, the labour process is over. And so you would stop the antibiotics at that point.” (ID06)
2.3 Caring for the mother–baby dyad in the current NHS maternity context	Expediting delivery of the baby as a treatment for suspected sepsis	27. I wouldn’t want to miss a septic lady or a septic baby. But then you see the only other treatment in labour is delivery. Get the baby out. Get the baby if that’s the source of the infection. Nine times out of 10, the treatment is deliver the baby. (ID24)
		28. I would say in my experience, probably over half of the women who have started on antibiotics for pyrexia in labour actually improve clinically, as soon as the baby’s born. (ID11)
		29. I think there are cons to giving antibiotics in the intrapartum phase where [you are] potentially creating an issue, and what ultimately definitively needs to be done is delivery. And that’s not necessarily about aiming for vaginal birth, it’s about identifying that there’s a tipping point, and where does that tipping point sit. If we get a baby out in good condition and they get no infection, but we’ve done a caesarean section, we’ve done the right thing or you know if you do it too late, should we have done it sooner. (ID16)
		30. “Its how near you are to delivery, so if you’re only two centimetres or something, it’s very different to being fully dilated and pushing the baby out because the antibiotics probably aren’t going to make any difference to the baby at that point. Whereas if you’ve you’re worried about infection early on in labour, that is going to make a difference to the baby. Whereas if you’re just giving them in as the baby is being pushed out, that’s going to have no impact on the baby. (ID07)
	Balancing outcomes for mums and babies	31. I think that sometimes we can have women who are starting to show a few signs of sepsis, but due to the woman’s wanting to try and get to viability or to have a live birth that we can sometimes all be persuaded to keep going with a situation that is potentially life threatening to the woman. (ID 02)
		32. “You’re exposing mum and potentially baby to medications that they didn’t perhaps need to be exposed to. And some of these antibiotics have unpleasant side effects, but also some risky side effects attached to them.” (ID06)
		33. Babies who do get unwell have significant consequences. So how many do you have to treat to impact that one baby? (ID07)
		34. “Bad outcomes do happen. You know, we might get away with it with mum and then find ourselves with a really sick baby, or a neonatal death. I think that’s the problem, isn’t it? That the stakes are, I certainly feel somewhat higher. (ID16)
	High stakes population to miss sepsis in	35. I think with anything within this sphere, it’s really difficult because the stakes are so high, you know sepsis can cause brain damage in babies. (ID22)
		36. I think with maternity because everyone is so fearful of everything that’s going on everywhere within the world of maternity the worry is if I miss those antibiotics and then that baby goes to NICU that will be my fault. So I think there’s a lot of, you know, defensive practice. (ID17)
		37. Well nobody wants to pick up next year’s M-BRRACE or in three years time pick up M-BRRACE and read a case report and go Yep, that was mine. Of course you don’t… We are all in this to do the best by women and their babies and their partners and their families… Pregnancy is a great confounder in terms of you can explain a lot by changes in physiology associated with the pregnant state… Unless there was an intervention, bedside test or quick lab result that was binary, sepsis, not sepsis, in this context, where the stakes are so, so high, I think it’s going to be hard to put that genie back in the bottle.” (ID20)
		38. It’s all the [maternity] investigations, isn’t it? I don’t think they perceive intrapartum antibiotics that is something they need to be worried about. (ID17)

NHS, National Health Service.

#### Subtheme 2.1: *the black-and-white of guidelines and the grey zone in practice*

All clinicians discussed sepsis guidelines as prescriptive pathways and flow charts with tick-box checklists that made decision-making less of a clinical judgement and more of a ‘black and white’ mandate; with starting antibiotics the default position (table 3, quotes 1–4). Using guidelines this way was viewed as helpful by, and safer for, doctors in training working across departments and Trusts. Guidelines were the ‘failsafe’, defensive strategy against missing sepsis. Some obstetricians thought Guideline tick-box checklists empowered midwives. Midwives had mixed views but said they did enable them to escalate for medical review and start antibiotics quicker. Most participants recognised nothing is black and white in practice, questioning the use of parameters from non-pregnant populations as red and amber flags for suspected sepsis during labour (table 3, quotes 5–8). Participants reported challenges stemming from the lack of an evidence base about normal ranges in pregnant labouring women for temperature, lactate, C reactive protein, and white cell count. In the absence of this evidence or a definitive test, clinicians had no alternative but to go with what is known about sepsis management from non-pregnant populations. Clinicians reported the dilemmas they faced when patients flagged as suspected sepsis based on single point-in-time observations and enacting the SEPSIS-SIX ran contrary to their clinical judgement (table 3, quotes 9–13).

#### Subtheme 2.2: *when to prescribe, when not and when to step down or stop*

Many clinicians discussed the challenges of trying to balance what could be normal observations for labour with what are also markers for developing sepsis to inform decision-making (table 3, quotes 14–18). Local guidelines that recommend using early warning scoring systems are key to deciding when to prescribe as part of the wider clinical picture. Clinicians described a delicate balance whereby clinicians think sepsis alongside hypothesising what is normal for this woman in this labour; is her respiratory rate explained by her labour progress (ie, pushing in second stage); her temperature raised because of her birth choices (ie, epidural for pain relief) or circumstance (ie, misoprostol for induction of labour). Bigger picture observations were particularly valued by senior midwives and experienced obstetricians (ie, does the woman look sick; is the baby tachycardic) alongside maternal obstetric early warning scores (Modified Early Obstetric Warning System, MEOWS). Variations in MEOWS triggers were reported across place and time, particularly for what counts as fever. Where there is doubt, the default is to prescribe because of the absence of quantitative measures to say what normal observations are for women during labour. This evidence gap sits alongside the absence of a perfect biomarker test for maternal sepsis during labour (subtheme 2.1). Junior doctors and midwives could not recall a situation where antibiotics were not given to women when a sepsis screen was triggered. Senior obstetricians recalled ‘really obvious’ situations, if abnormal heart rate and blood pressure observations were attributable to another cause (ie, postpartum haemorrhage), that a decision not to prescribe could be made (table 2, quote 22). Guidelines advocate early escalation to seniors because pregnant women can deteriorate quickly. Some consultants described using their clinical acumen less when to prescribe, and more in decision-making about when to step down or stop (table 3, quotes 23–26).

#### Subtheme 2.3: *caring for the mother–baby dyad in the current NHS maternity context*

Senior clinicians and midwives described how, when chorioamnionitis is suspected, delivery should be expedited. The decision to deliver was viewed as a tipping point (table 3, quotes 27–31). Women with preterm prelabour rupture of membranes, particularly early before or at the limits of viability, challenged decision-making. Obstetricians and midwives described how these women are most at risk of developing and dying from sepsis, but the least likely to want to be delivered. Clinicians’ decisions were impacted by the need to balance maternal and fetal outcomes. (table 3, quotes 32–34). While clinicians described their principal focus as the mother–baby dyad in front of them, they were also influenced by the current spotlight on the quality of NHS maternity care in the UK. This weighed on many clinicians’ minds. Mothers and babies are a high-stakes population and any risks of adverse reactions or other negative effects associated with antibiotics were outweighed by the benefits of not missing sepsis for the baby, mother and clinician (table 3, quotes 35–38). One clinician suggested care is more “*interventional for everybody because of that idea that we don’t want to look like we’ve missed anybody who is a case of sepsis”* (ID02).

### Theme 3: clinicians’ views about the impact of their decision-making: ‘*If we continue down this path where might we be?’*

Themes 1 and 2 show that conflating infection and suspected sepsis with severe sepsis can make clinicians feel compelled to prescribe broad-spectrum antibiotics to prevent poor outcomes for individual mothers and babies, and themselves. This theme reports what clinicians think about the impact of their decision-making for population health and where the bigger picture problems lie when priorities for sepsis management and AMR are linked. Clinicians identified sepsis management impacts women’s experiences, changes workloads and could negatively contribute to AMR. The issues with current sepsis management are encapsulated in the quote below:

*The much harder kind of harm to see is the big picture stuff that we’ve spoken about in terms of [AMR] that’s the tricky thing and lots of areas of obstetrics it is easy to look back at a kind of single adverse outcomes like a post-natal readmission with the mindset, you know, with an infection and things “Oh, why didn’t we? You know, we should just be giving all these women antibiotics”, but actually that’s because they’re not measuring things like, neonatal microbiome problems, antimicrobial resistance at a regional or national level. We’re not. We’re not looking at that data. So can we really see that flip side of it. [ ] Where we are now… clearly it’s only a matter of time before we do see serious morbidity and mortality related to those bugs themselves… [ ] And I think if we, if we just continue laying down this path, we’re gonna end up with even more problems. It’d be good to kind of stop and check where we are and maybe fill in some of the gaps. (Obstetrician, ID05)*

Senior clinicians recognise AMR will impact the care of women during labour, the safety of instrumental and caesarean birth, and care of newborns in the future. However, AMR is set to remain a lesser priority than rapid sepsis management in the national maternity safety agenda until better evidence for management in labour exists. An overview of subthemes, codes and illustrative quotes can be found in table 4.

**Table 4:** Clinicians’ views about the impact of their decision-making

Subtheme	Codes	Supporting quotes
3.1 Impacts women’s experiences	Side effects for mums and babies	1. “You’re exposing mum and potentially baby to medications that they didn’t perhaps need to be exposed to and some of these antibiotics have unpleasant side effects, but also some risky side effects attached to them. Anaphylaxis would be the big one. She [mum] develops anaphylaxis, then you’ve got a baby to think about as well. But all the other things, diarrhoea, nausea, and vomiting and just generally feeling quite rubbish if you’re giving it IV. Some of these antibiotics can be, they can sting and burn as they go through a drip.” (ID06)
		2. “The main disadvantages if they don’t need them is longer hospital stay, increased anxiety and stress, possibly additional monitoring of baby or if, depending on the hospital if mum’s been on antibiotics, they automatically do put baby on antibiotics. I had one person who did have an infection who it’d been documented she had an allergy. For some, it can just have side effects of diarrhoea and vomiting.” (ID13)
		3. “You’re going to expose an individual to potential allergen. So they could have reactions to antibiotics that perhaps they didn’t need.” (ID08)
		4. “In the first few hours after baby’s born you are separating mum and baby at a time when colostrum’s coming, skin-to-skin is very important, bonding. You’re causing psychological upset. [ ] I think there’s also a growing body of evidence my understanding is about antibiotics and the impact that it has on the microbiome, both of a newborn baby and of mum.” (ID02)
	Catalyst for a cascade of interventions for mum and baby	5. “I do lots of debrief appointments and women will often describe, what is it they say oh, how do you describe a something of interventions, cascade of interventions, yes, they’ll often describe, they thought I had an infection, so next thing I’m having blood taken and catheters and you know, (…) Multiples of women who come back to you and say (…) labour put them in danger, this sense they were in danger essentially is what they get from that sepsis effect. That has a PTSD trauma-type effect on them.” (ID04)
		6. When mums had antibiotics, the baby then needs to go for a septic screen. Nobody likes the thought of their baby having to go have blood tests, heel prick test, antibiotics, a cannula. All of this seems quite invasive, often leads to a longer hospital stay with the need for antibiotics for the baby and the ongoing care. (ID02)
		7. “She needs a drip to have them put in, which she may otherwise have not needed to have.” (ID06)
		8. “I think it probably does impact caesarean and instrumental rate, definitely.” (ID21)
	Subsequent pregnancies	9. “I think it gets to the point where they cannot consider a vaginal birth now because they’re worried about the impact of infection and all those sorts of things (…) they’re more likely to choose an elective section, next time, and therefore those people will get antibiotics and it all kind of feeds back into it as well. And I think the awareness around birth trauma is increasing as well. And I think part of you know, developing an infection is seen as like an adverse event. And therefore that will have probably made some women question whether they want to consider a vaginal birth again if they’ve ended up with an infection from it.” (ID12)
		10. “The group of women who are perhaps traumatised by being told that they had sepsis when actually they just had a good progressing labour they go on and have a spontaneous vaginal birth but they’ve heard the word sepsis. They’re kind of traumatised, that group of women, going on to have a caesarean section, a planned caesarean section the next time which is not good, is not good, for anybody. So these are women who’ve had a vaginal birth, and are likely to have a low-risk birth the second time with lower complications (…) it’s better for everybody if somebody’s had a previous vaginal birth goes on and has another vaginal birth but these are groups of women who are traumatised by the language that we may have used in their first labour.” (ID04)
		11. “Women who I’ve not looked after previously but have previously had a labour, laboured and had antibiotics, baby therefore had to get a course so they’re in for another at least 24 hours and then they come back, on my section list and say well, actually, you know, I’ve had all of that and I just didn’t want any of it this time so that’s why I’ve elected to have a planned caesarean. I mean, my main area of interest is the absolute skyrocketing and, you know, tsunami of planned sections we’re going to be doing in the next 10 years. A lot of that, a lot of women are post-Montgomery choosing, certainly, even people who have had previous vaginal deliveries to go for a section, particularly if they’ve had issues around peripatum period and an extra 24 hours in hospital with baby receiving antibiotics is, you know, a potential thing that women, I think it would play into women’s decision making about whether they wanted to be labelled with sepsis or not.” (ID20)
3.2 Changing workloads	Changing workloads-sepsis management antibiotics use as the norm	12. “I feel like there’s kind of an over-awareness. I think it’s important because true sepsis killed people and so it’s important that the awareness is there, identifying it because early intervention actually prevents that. But I feel now it’s become a grey area of this person has sepsis. This person has sepsis this is actual sepsis. It’s just, it seems it’s too like in a way, blase. I mean, I am probably part of that problem. I use the term sepsis because it’s more, it’s more like if I prescribe antibiotics because of that, it seems more like oh, I can justify it.” (ID13)
		13. “The ANODE Trial (of instrumental deliveries) came out when I was only a second-year trainee in Obs & Gynae… we’ve always given antibiotics for caesareans since I’ve practised. So I think that we're just quite used to giving antibiotics in labour.” (ID02)
		14. “On labour ward people joke everybody gets suspected maternal sepsis. All these inductions [of labour]… it’s this kind of Stockholm syndrome we’ve given you sepsis and now we’re going to save you from it.“ (ID22)
		15. “We do a lot more inductions of labour than we used to.” (ID24)
	Changing workloads–AMR now	16. “I don’t think the juniors [doctors] and the midwives think of the consequences of overprescribing antibiotics, which would worry me perhaps more because I get, I’m the person having to deal with the resistant infections, they’re the what are you're going to do now that happens to the seniors.” (ID17)
		17. “If you look at the big picture of things in terms of antimicrobial resistance, certainly in our unit we’re dealing with, where it’s much more common now that we’re getting resistant, resistant organisms even in a relatively well population who probably don’t have a lot of prior antimicrobial kind of exposure. But we’re still getting, and particularly as we’re getting people from other areas other countries or have been in hospitals in other regions, you know, we're getting a lot more resistant organisms. And that has quite a big impact on the care that we can provide to everyone, not just from our kind of Health Protection point of view, but actually capacity in the unit and all these other things because you’re having to put in such stringent measures to avoid kind of transmission of these things.[ ] When you have someone (known resistant), it creates a huge amount of strain [ ] your kinda running around trying to isolate them, give them quite complicated antimicrobial treatment to get it sorted. So I guess my concern is that’s where we are now.” (ID05)
		18. “It [AMR] becomes quite a headache for micro. I would say you know regionally there are different bugs in different populations, so looking nationally, it’s very difficult to say one antibiotic covers all because we know that’s not true.” (ID08)
	Changing workloads–AMR in the future	19. “If there’s less and less antibiotics available to treat, then that eventually poses a risk to the mothers and babies of the future. If there’s none left anymore to treat those that have real, proper infection, which there will be of course, because we know there are certain infections that absolutely kill mothers and others that kill babies.” (ID03)
		20. “So the more people are exposed to broad-spectrum antibiotics, the more they might, bugs might develop resistance to it, and then we develop these super bugs that we can’t treat. There are already, obviously numerous cases of that. The issue if you get antibiotic resistant bugs, as we know, is these bugs can lead to premature labour. So if they become harder to treat, and then perhaps the risk of premature labour, especially if people get those bugs in the future.” (ID08)
		21. “If you start to go the other way, where you’re not raising the awareness [of sepsis] or you’re making barriers that kind of could delay, then you could really see that increase in poor outcomes like mortality. But then I don’t know, I haven't come across any patients with resistance.” (ID13)
3.3 AMR and sepsis is the future	Here and now, AMR and antimicrobial stewardship are secondary priorities to sepsis	22. “Sepsis is a word that is understood and known a lot more now. I’m not sure if antimicrobial resistance has somehow made it into people’s brains so much.” (ID02)
		23. “In obstetrics, we’re kind of living with a kind of big push on treating sepsis. But actually It’s just frustrating that the last kind of 10–15 years we’ve not really done anything, it’s just been this big push and then there’s no actual evidence kind of filling in behind it to inform where we go from here.” (ID05)
		24. “There are societal downsides of overprescribing of antibiotics in general, indiscriminate use of antibiotics is not good in the long term.” (ID01)
		25. “I feel like we do think about anti microbial resistance, but that’s kind of more antibiotics, wider prescriptions during pregnancy, the query sepsis in labour, it’s just to follow the guideline. [ ] So it’s kind of the decision about worrying about resistance is taken out of your hands, if that makes sense”. (ID09)
		26. “Between the individual and the public health element of things, the negative side to giving them antibiotics is antibiotic resistance” (ID17).
	Impact of present antimicrobial use on next generation	27. “It’s probably a majority of women now who get antimicrobials around the time of birth. Lets’s face it, if our section rate is 30–35%, we have a reasonable number of women who are GBS positive who will get antibiotics in labour, we have an instrumental delivery rate of around 15% and those women should in theory have some antibiotics as per the ANODE trial, at least a single dose of broad-spectrum antibiotics. So you’re already at over 50% of your of population getting antibiotics around the time of birth. I just don’t know if that’s the right thing. [ ] I think there’s the bigger unknown about what does it, you know, what impact does it have the neonatal microbiome or foetal neonatal microbiome and those things.” (ID05)
		28. “Many more people are getting antibiotics than perhaps did do before. Pre-term births too. So many more people are getting antibiotics and perhaps they did do when I started in 2010. All the Caesareans. Mums get antibiotics as prophylaxis for their surgery. (…) I don’t know the data but the concern is if you’re exposing new babies to the antibiotics and changing their, the microbiome, that they’ve just acquired from their mother by caesarean or normal delivery, normal birth then that might have some childhood effect on their immune system that we’re yet to quantify.” (ID10)
	AMR is a bigger problem than maternity	29. “If you go to my GP surgery there are plenty of posters and I think that people often know if I say if I speak to them about finishing the course, they know that that’s important.” (ID02)
		30. “I think globally. I think [in UK] GP’s prescribe far too much. You know, I think it’s more of an issue in GP land and A&E. Just people being, it’s the culture, isn’t it, patient expectation as a society that they go to the GP with a cough and they want antibiotics.” (ID07)
		31. “You have to remember that there are a lot of countries where people can just buy, and have bought, antibiotics over the counter. That’s much more dangerous… It needs to come from WHO or something like that but then that only works if governments take that on. And their laws, I suppose, like we have here in the UK and most of the European countries… And that system change. I feel countries, certain countries that have that knowledge (antibiotic misuse, AMR cases leading to deaths), should shout on the rooftops. [ ] It probably does need to be in the media because it’s only if there’s a media uproar about it that people take note.” (ID03)

AMR, antimicrobial resistance; NHS, National Health Service.

#### Subtheme 3.1: *women’s experiences*

Many clinicians expressed concerns that low thresholds for treatment of suspected sepsis expose mums and babies to antimicrobials. Anaphylaxis was the side effect that most participants expressed concern about (table 4, quotes 1–4). Some clinicians talked about the unpleasantness of diarrhoea, nausea and vomiting, stinging and burning during IV administration. Women’s experiences were felt to be negatively impacted as the sepsis pathway can also be the catalyst for other interventions, including instrumental and emergency caesarean birth. Babies may also require further intervention following birth which impacts on bonding and breastfeeding, as babies may require neonatal admission or screening resulting in separation from the mother (table 4, quotes 5–8). Several participants linked potential overtreatment with birth trauma. The impact was thought to extend beyond the current pregnancy. Participants suggested that women who were unwell with sepsis may not plan to have another baby. Midwives and obstetricians involved in postnatal debriefing services suggested sepsis pathways contributed to birth trauma among women who did not have sepsis. Consultant obstetricians and anaesthetists said women who had previously laboured and had antibiotics are increasingly electing for a planned caesarean birth in subsequent pregnancies (table 4, quotes 9–11).

#### Subtheme 3.2: *changing workloads*

Clinician’s described changing workloads associated with increasing numbers of women on sepsis pathways (table 4, quotes 16–18). The bulk of this work fell to midwives and trainee doctors, but it frequently requires senior input too. Broad-spectrum antibiotic prescribing for suspected sepsis was described as so common that it has become the norm on every shift. Senior clinicians were more concerned about the impact of AMR now, because they dealt with cases. Whereas junior trainees were least likely to link sepsis and AMR, or worry about stewardship. There were concerns about the future from a few clinicians who said maternity could reasonably expect a return to much higher death rates from infection because antibiotics are currently relied on for prevention of poor outcomes in preterm rupture of membranes, and all instrumental and caesarean births (table 4, quotes 19–21).

#### Subtheme 3.3: *AMR is the future*

Clinicians focused on the current care episode. AMR and antimicrobial stewardship are secondary priorities to sepsis or not a priority at all. Some felt following the guidelines absolved them of any responsibility for overtreatment (table 4, quote 25). Others thought the risk–benefit ratio was so weighted in favour of preventing sepsis that any downstream consequences were irrelevant. Many clinicians mentioned the effects of antimicrobials on the baby’s microbiome, and some were particularly concerned for the next generation, most of whom will have been exposed in utero (table 4, quotes 27–28). Clinicians recognised AMR is a bigger problem than maternity. Some mentioned overprescribing in primary care, others worried the internet has an unregulated supply of antibiotics, and a few said global travel and migration mean AMR requires an international response (table 4, quotes 29–31).

**Figure 3:**
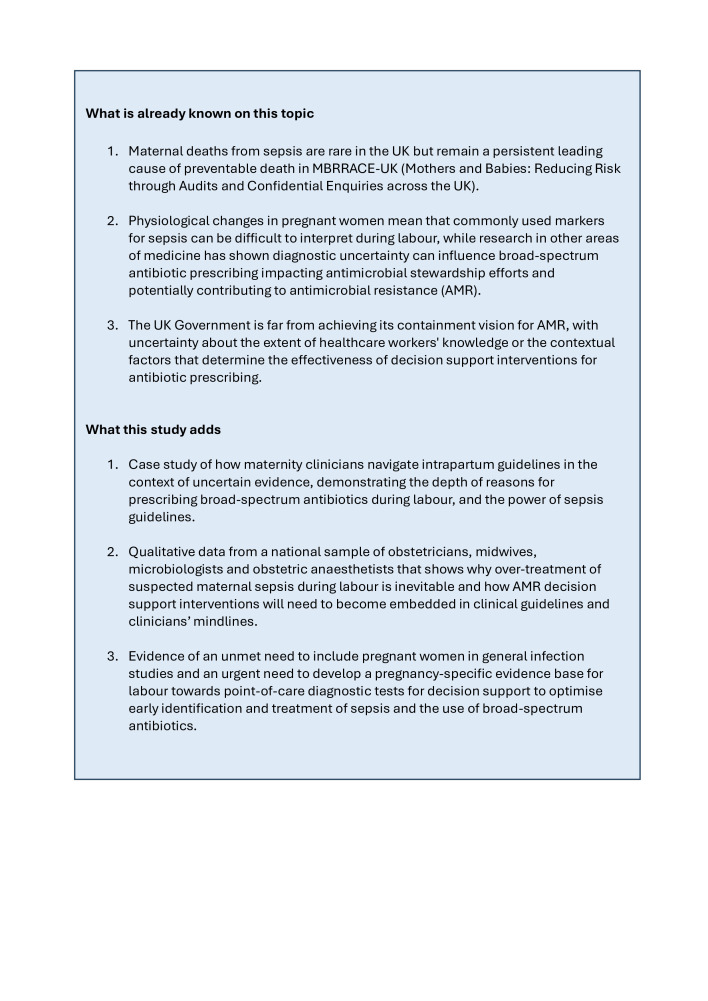
Key points. AMR, antimicrobial resistance; MBRRACE, Maternal, Newborn and Infant Clinical Outcome Review Programme.

A summary of findings is provided in figure 3.

## Discussion

This study shows how clinicians make prescribing decisions for suspected maternal sepsis during childbirth, offering a novel framework to explain why overtreatment can happen. Just over a third of participants believed overprescribing is occurring and is a problem. Most thought overprescribing might happen, but did not perceive it as a problem. Clinicians reported a high index of suspicion for sepsis and feeling compelled to initiate broad-spectrum antibiotic treatment pathways. A lack of evidence from pregnant populations underpinning guidance, paradoxically increases reliance on guidelines to counter-balance diagnostic uncertainty. The absence of evidence, clinical tools and diagnostics to distinguish physiological labour from developing sepsis which are acceptable and accessible makes overtreatment of suspected sepsis inevitable. Clinicians fear missing sepsis and collectively reinforce the idea not to give antibiotics is brave. Diagnostic uncertainty and high stakes, mean clinicians need guidelines to defend actions and direct decision-making. We also report clinicians’ views about the impact of their decision-making to the linked priorities of sepsis management and AMR. The risk of harm versus the benefits of antibiotics was perceived to be low by some participants, but not all. Clinicians report antibiotic-associated adverse effects and antibiotic resistance. Population-level surveillance data for sepsis or AMR were not widely known, the societal risks are hard to quantify, and their priority is their patient(s). Paying attention to maternity clinicians’ mindlines (meaning embodied clinical wisdom, collectively enforced) identified important gaps in the evidence, and challenges for clinicians providing care in these circumstances. To the best of our knowledge, this is the first study to apply the concept of mindlines in intrapartum care and address the linked national priorities of early sepsis management and AMR.

### What this study adds in relation to existing research

The key strength of this study is evidencing how maternity clinicians navigate intrapartum guidelines in the context of uncertain evidence, demonstrating the depth of reasons for prescribing broad-spectrum antibiotics during labour, and the power of sepsis guidelines. In so doing, it transcends dichotomising overtreatment using broad-spectrum antibiotics for suspected maternal sepsis as at odds with the early identification and treatment of sepsis to reveal the nuances in the process of care, the blind spots in individual practice, and the tensions in the system, which interventions to address AMR and sepsis management as linked priorities must transcend. In the UK, a third national ambition exists alongside the visions for sepsis and AMR, which is the national maternity safety ambition that aims to halve the rates of stillbirths, neonatal and maternal deaths, and brain injuries.^52^ We offer an illustrated thematic framework of clinicians’ mindlines because this knowledge-in-practice-in-context may play an inescapable role in developing good clinical care.^36^ We show mindlines can aid understanding of both the strengths in existing practice (ie, when to step down broad-spectrum antibiotics) and the gaps in the evidence base (ie, decision support tools that can distinguish between normal physiological processes and sepsis to better support decisions of when to start broad-spectrum antibiotics); the key step towards filling them.

This study also adds to, but differs from, existing research concerned with guideline non-adherence.^53^ Global systematic reviews show intrapartum guideline use is not ubiquitous. Uptake varies according to guideline topic and scope, clinicians’ personal values, professional experience, beliefs about underpinning evidence, education and training, resources and health system.^54 55^ Our findings do align with existing research from other medical specialities investigating infection, suspected sepsis and sepsis concerning the role of language in clinicians’ conversations about sepsis and how conflating infection, suspected sepsis and sepsis has consequences for care^17 44^; and how in situations of diagnostic uncertainty, ICU clinicians will overtreat to protect patients from the possibility of deterioration and protect themselves from the ethical and legal consequences.^18^ Ensuring timely identification and management of maternal sepsis while addressing AMR does require a multifaceted approach.^30 31^

For maternity, we show that decision-support interventions will need to allay safety concerns for the mother–baby dyad. Research is emerging to advance the management of maternal, fetal and neonatal infection using point-of-care tests,^34^ including first in pregnancy testing,^56^ participants in this study would welcome this step. Our reporting coincides with the call from the INHALE Trial team for a ‘technology plus’ approach that recognises the challenges clinicians face when applying technological solutions to patient care.^42^ Although the focus of INHALE was infection, not suspected sepsis per se, they conclude technological and guideline solutions to AMR will be limited if we fail to recognise the impact of clinical mindlines on prescribing decisions. In INHALE, mindlines were key drivers of antibiotic prescribing above hospital prescribing guidelines and the results of molecular diagnostics. Our findings add weight to their call and show how mindlines constructed through successive sepsis campaigns and guidelines will need to be reformed through an implementation ‘technology plus’ approach. It is not enough to have evidence underpinning clinical guidelines; it must be in clinicians’ mindlines too.

### Strengths and limitations

The findings of this study are not intended to be generalisable. However, the qualitative data we report may be transferable,^57^ resonating with clinicians’ views and experiences beyond the context in which this study was conducted. A key strength of this study is its interdisciplinary approach, combining medical and social science knowledge claims in a qualitative methodology. The sample size was sufficient to claim theme and meaning saturation and adheres to current methodological guidance.^46^ We used the Framework Approach to thematic qualitative analysis to enhance trustworthiness. We used deductive and inductive coding, with the latter leading to a refinement of the thematic framework to include the impact of clinicians’ decision-making. Weaknesses include only three midwife participants (from North, South and Midlands), and the absence of patient and public views, although beyond the scope of this study.

### Implications for clinicians and policy makers

AMR has the potential to make sepsis much more difficult to treat. This study suggests gaps in clinicians’ knowledge of interventions and outcomes in the management of suspected infection. There may be scope for introducing practice-based learning about suspected sepsis practice and outcomes, linked with AMR reporting. Improving awareness of national data, for example, the English surveillance programme for antimicrobial utilisation and resistance,^58^ together with Maternal, Newborn and Infant Clinical Outcome Review Programme^4^ and NHS Maternity statistics, will enable clinicians to contextualise their actions.^59^ Clinicians in this study were acutely aware of the impact of the media and public understanding. Policymakers should note that the success of the sepsis campaigns has been in the longevity of consistent and concise messaging, which AMR campaigns could learn from, towards the UK’s containment vision.^32 33^ Any future public campaign will need strong evidence underpinning it, which clinicians believe in alongside a concerted effort to change public opinion.

### Unanswered questions and future research

This study leaves unanswered questions. There is an urgent need to develop the evidence base for biomarkers, physiological parameters and diagnostics which can be applied during labour, to underpin clinical decision support tools. Faster, better and earlier diagnosis was a theme in The Future State of Health and Healthcare in 2035 report, compiled for the new NHS 10-Year Plan. Improved diagnostics (including pathogen-based tests, biomarkers and data that aid in making a diagnosis, eg, sepsis) are crucial for tempered decision making.^60^,^62^ The NICE guideline’s research recommendations include the need to develop a set of clinical decision rules or a predictive tool for sepsis, but there is no mention of research involving pregnant women.^28^ Not prioritising research for pregnant, labouring women disadvantages women and their infants everywhere.^63^ In the UK context, where risk factors for severe sepsis include ethnicity and deprivation, with black and other ethnic minority groups at an almost twofold increased risk of maternal death, not investigating decision rules for pregnant women and debiasing research designs to include the most vulnerable is a double disadvantage. It runs contrary to the Women’s Health Strategy,^64^ NHS CORE20+5 action to reduce inequalities,^65^ and NICE and the NHS Race & Health Observatory’s agreement to tackle health inequalities.^66^

The current national action plan (2024–2029) towards the UK 20-year vision for AMR seeks to support frontline clinicians in making the best choices regarding antimicrobial treatments with decision support and risk stratification tools, alongside appropriate diagnostic tests.^13 14 32 33^ This could support maternity clinicians in their decision-making; however, it would need to be underpinned by an improved understanding of usual labour physiology and diagnostic tests will need to be specifically validated. With pregnant women and newborns at higher risk of sepsis, there is an urgent need to expedite research to advance the management of maternal, fetal and neonatal infection. This study adds support to the need to include pregnant women in general infection studies and an urgent need to develop a pregnancy-specific evidence base for labour towards point-of-care diagnostic tests for decision support to optimise early identification and treatment of sepsis and the use of broad-spectrum antibiotics.

## Conclusions

We conclude that the overtreatment of suspected maternal sepsis during labour may be inevitable. Clinicians prioritise the mother–baby dyad in front of them, and therefore, antimicrobial stewardship comes second to concerns about missing sepsis. Sepsis prevention, identification and treatment dominate clinicians’ mindlines. To address overtreatment during labour, clinicians will require better evidence to support decision-making about what is a normal physiological process, and when urgent treatment is required to prevent sepsis. Overtreatment of suspected maternal sepsis is likely until point-of-care diagnostic tests, supported by high-quality decision tools, exist for women in labour. Implementation considerations for any such innovation will require a ‘technology plus’ approach towards adoption, meaning it is implemented in guidelines and mindlines.

## Supplementary material

10.1136/bmjopen-2025-110559 online supplemental file 1

10.1136/bmjopen-2025-110559 online supplemental file 2

10.1136/bmjopen-2025-110559 online supplemental file 3

## Data Availability

All data relevant to the study are included in the article or uploaded as supplementary information.

## References

[1] World Health Organization (2017). Statement on maternal sepsis. https://www.who.int/publications/i/item/WHO-RHR-17.02.

[2] Chen L, Wang Q, Gao Y (2021). The global burden and trends of maternal sepsis and other maternal infections in 204 countries and territories from 1990 to 2019. BMC Infect Dis.

[3] Say L, Chou D, Gemmill A (2014). Global causes of maternal death: a WHO systematic analysis. Lancet Glob Health.

[4] MBRRACE-UK Maternal, Newborn and Infant Clinical Outcome Review Programme Saving lives, improving mothers’ care. Lessons learned to inform maternity care from the UK and ireland confidential enquiries into maternal deaths and morbidity 2020-22.

[5] O’Higgins AC, Egan AF, Murphy OC (2014). A clinical review of maternal bacteremia. Intl J Gynecol Obste.

[6] Shah NM, Charani E, Ming D (2023). Antimicrobial stewardship and targeted therapies in the changing landscape of maternal sepsis. J Intensive Med.

[7] Acosta CD, Kurinczuk JJ, Lucas DN (2014). Severe maternal sepsis in the UK, 2011-2012: a national case-control study. PLoS Med.

[8] Acosta CD, Harrison DA, Rowan K (2016). Maternal morbidity and mortality from severe sepsis: a national cohort study. BMJ Open.

[9] National Institute for Health and Care Excellence (2024). NG51 *suspected sepsis: recognition, diagnosis and early manage*ment (B) *evidence review for managing and treating suspected sepsis in acute hospital settings; antibiotic treatment in people with suspected s*epsis.

[10] Schorr CA, Dellinger RP (2014). The Surviving Sepsis Campaign: past, present and future. Trends Mol Med.

[11] Lin Y (2021). Effectiveness of the sepsis six bundle in the management of acute adult sepsis in the UK. Emerg Nurse.

[12] GBD 2021 Antimicrobial Resistance Collaborators (2021). Global burden of bacterial antimicrobial resistance 1990-2021: a systematic analysis with forecasts to 2050. Lancet.

[13] Tang KWK, Millar BC, Moore JE (2023). Antimicrobial Resistance (AMR). Br J Biomed Sci.

[14] HM Government (2024). Confronting antimicrobial resistance 2024 to 2029. https://assets.publishing.service.gov.uk/media/664394d9993111924d9d3465/confronting-antimicrobial-resistance-2024-to-2029.pdf.

[15] HM Government (2019). Contained and controlled: the UK’s 20-year vision for antimicrobial resistance.

[16] Burston J, Adhikari S, Hayen A (2017). A Role for Antimicrobial Stewardship in Clinical Sepsis Pathways: a Prospective Interventional Study. Infect Control Hosp Epidemiol.

[17] Armstrong N (2018). Overdiagnosis and overtreatment as a quality problem: insights from healthcare improvement research. BMJ Qual Saf.

[18] Pandolfo AM, Horne R, Jani Y (2022). Understanding decisions about antibiotic prescribing in ICU: an application of the Necessity Concerns Framework. BMJ Qual Saf.

[19] Abutheraa N, Grant J, Mullen AB (2020). An Observational Cohort Study Evaluating Antimicrobial Use in Peripartum Sepsis: A Tendency towards Overdiagnosis?. Pharmacy (Basel).

[20] Acosta CD, Knight M, Lee HC (2013). The continuum of maternal sepsis severity: incidence and risk factors in a population-based cohort study. PLoS One.

[21] Greenfield B (2025). Audit of lactate in labour.

[22] Royal College of Obstetricians and Gynaecologists (2012). Sepsis in pregnancy, bacterial (green-top guideline no.64a).

[23] Royal College of Obstetricians and Gynaecologists (2012). Bacterial sepsis following pregnancy (green-top guideline no.64b).

[24] Royal College of Obstetricians and Gynaecologists (2024). Identification and management of maternal sepsis during and following pregnancy (green-top guideline no. 64). https://www.rcog.org.uk/guidance/browse-all-guidance/green-top-guidelines/identification-and-management-of-maternal-sepsis-during-and-following-pregnancy-green-top-guideline-no-64/.

[25] National Institute for Health and Care Excellence (2016). Sepsis: *recognition, diagnosis and early manag*ement. NG51.

[26] Bauer ME, Balistreri M, MacEachern M (2019). Normal Range for Maternal Lactic Acid during Pregnancy and Labor: A Systematic Review and Meta-Analysis of Observational Studies. Am J Perinatol.

[27] Dockree S, O’Sullivan J, Shine B (2022). How should we interpret lactate in labour? A reference study. BJOG.

[28] National Institute for Health and Care Excellence (2024). Overview | suspected sepsis: recognition, diagnosis and early management | guidance.

[29] National Institute for Health and Care Excellence (2015). Overview | antimicrobial stewardship: systems and processes for effective antimicrobial medicine use | guidance.

[30] Holmes AH, Moore LSP, Sundsfjord A (2016). Understanding the mechanisms and drivers of antimicrobial resistance. The Lancet.

[31] Hulscher MEJL, Grol RPTM, van der Meer JWM (2010). Antibiotic prescribing in hospitals: a social and behavioural scientific approach. Lancet Infect Dis.

[32] National Audit Office (2025). Investigation into how government is addressing antimicrobial resistance. https://www.nao.org.uk/press-releases/government-a-long-way-from-achieving-its-vision-of-containing-antimicrobial-resistance/#report.

[33] Wilkinson E (2025). Efforts to tackle antimicrobial resistance are having “limited impact,” NAO warns. *BMJ*.

[34] Ming DK, Merriel A, Freeman DME (2024). Advancing the management of maternal, fetal, and neonatal infection through harnessing digital health innovations. Lancet Digit Health.

[35] O’Brien BC, Harris IB, Beckman TJ (2014). Standards for reporting qualitative research: a synthesis of recommendations. Acad Med.

[36] Gabbay J, Le May A (2023). Knowledge *transformation in health and social care: putting mindlines to wo*rk.

[37] Gabbay J, le May A (2004). Evidence based guidelines or collectively constructed “mindlines?” Ethnographic study of knowledge management in primary care. BMJ.

[38] Gabbay J, Ie May A (2011). Practice-based evidence for health care: clinical mindlines.

[39] Wieringa S, Greenhalgh T (2015). 10 years of mindlines: a systematic review and commentary. *Implementation Sci*.

[40] Chandler CIR, Jones C, Boniface G (2008). Guidelines and mindlines: why do clinical staff over-diagnose malaria in Tanzania? A qualitative study. Malar J.

[41] Grant A, Sullivan F, Dowell J (2013). An ethnographic exploration of influences on prescribing in general practice: why is there variation in prescribing practices?. Implement Sci.

[42] Stewart S-JF, Pandolfo AM, Jani Y (2025). Guidelines vs mindlines: a qualitative investigation of how clinicians’ beliefs influence the application of rapid molecular diagnostics in intensive care. *Antimicrob Agents Chemother*.

[43] Olmos-Vega FM, Stalmeijer RE, Varpio L (2023). A practical guide to reflexivity in qualitative research: AMEE Guide No. 149. Med Teach.

[44] Kingdon C (2005). Reflexivity: Not just a qualitative methodological research tool. Br J Midwifery.

[45] Gentles S, Charles C, Ploeg J (2015). Sampling in Qualitative Research: Insights from an Overview of the Methods Literature. TQR.

[46] Wutich A, Beresford M, Bernard HR (2024). Sample Sizes for 10 Types of Qualitative Data Analysis: An Integrative Review, Empirical Guidance, and Next Steps. Int J Qual Methods.

[47] Heckathorn DD (2011). Snowball versus respondent-driven sampling. Sociol Methodol.

[48] Hennink M, Kaiser BN (2022). Sample sizes for saturation in qualitative research: A systematic review of empirical tests. Soc Sci Med.

[49] Carmel S, Jacobi E (2024). Exploring valuation practices in diagnosis-as-category: The rising dominance of clinical practice in the categorisation of Sepsis, 1991-2016. Sociol Health Illn.

[50] Ritchie J, Spencer L, Bryman A, Burgess RG (1994). Analyzing qualitative data.

[51] Gale NK, Heath G, Cameron E (2013). Using the framework method for the analysis of qualitative data in multi-disciplinary health research. BMC Med Res Methodol.

[52] Department of Health (2017). Safer maternity care: the national maternity safety strategy - progress and next steps.

[53] Lange S, Mwisongo A, Mæstad O (2014). Why don’t clinicians adhere more consistently to guidelines for the Integrated Management of Childhood Illness (IMCI)?. Soc Sci Med.

[54] Kingdon C, Downe S, Betran AP (2018). Interventions targeted at health professionals to reduce unnecessary caesarean sections: a qualitative evidence synthesis. BMJ Open.

[55] Kingdon C, Downe S, Betran AP (2018). Non-clinical interventions to reduce unnecessary caesarean section targeted at organisations, facilities and systems: Systematic review of qualitative studies. PLoS One.

[56] Freeman D First in pregnancy continuous lactate monitoring using a minimally invasive microneedle device: a proof-of-concept study in healthy pregnant volunteers.

[57] Lincoln YS, Guba EG (1985). Naturalistic *i*nquiry.

[58] HM Government English surveillance programme for antimicrobial utilisation and resistance (ESPAUR) report - GOV.UK. https://www.gov.uk/government/publications/english-surveillance-programme-antimicrobial-utilisation-and-resistance-espaur-report.

[59] NHS England Digital NHS maternity statistics England 203-24. https://digital.nhs.uk/data-and-information/publications/statistical/nhs-maternity-statistics.

[60] Horner B, Yong C, Chen E (2025). Better & earlier diagnosis. https://www.imperial.ac.uk/media/imperial-college/institute-of-global-health-innovation/centre-for-health-policy/public/Theme-2-Faster,-Better-&-Earlier-Diagnosis.pdf.

[61] Centre for Health Policy (2025). Future state of healthcare. https://www.imperial.ac.uk/centre-for-health-policy/our-work/digital-health/future-state/.

[62] HM Government (2025). 10 year health plan for England: fit for the future - GOV.UK.

[63] Dey T, Widmer M, Coomarasamy A (2025). Advancing maternal and perinatal health through clinical trials: key insights from a WHO global consultation. Lancet Glob Health.

[64] Department of Health and Social Care (2022). Women’s health strategy for England - GOV.UK.

[65] NHS England Core20PLUS5 (adults) – an approach to reducing healthcare inequalities.

[66] NHS Health and Race Observatory (2025). Observatory signs unique partnership agreement with NICE to tackle inequalities in health - NHS – Race and Health Observatory.

