# Supplementary material for: Clinicians’ decision-making about broad-spectrum antibiotic prescribing for suspected maternal sepsis during childbirth in the UK: a qualitative study
Source: BMJ Open. 2026 Jul 17;16(7):e110559. doi: 10.1136/bmjopen-2025-110559 (PMC13384126; doi:10.1136/bmjopen-2025-110559)
Supplement: online supplemental file 1 [file bmjopen-16-7-s001.docx]

**Supplementary Table 1: SRQR Checklist**

| **No.** | **Topic** | **Item** | **Page** |
| --- | --- | --- | --- |
| **Title and abstract** | | |  |
| S1 | **Title** | Identifies the study as a qualitative study | 1 |
| S2 | **Abstract** | Summary of qualitative design, method and data analysis. | 2 |
| **Introduction** | | |  |
| S3 | **Problem formulation** | Description of maternal sepsis, antimicrobial resistance (AMR) and over-prescribing in other medical specialities and maternity. | 4 |
| S4 | **Purpose or research question** | This study aimed to explore views and decision-making experiences of doctors and midwives about broad-spectrum antibiotic prescribing for suspected maternal sepsis in women admitted to hospital for childbirth, to identify if, how, and why over-treatment may occur. | 4 |
| **Methods** | | |  |
| S5 | **Qualitative approach and research paradigm** | Qualitative study using semi-structured interviews. Underpinning theory is Gabbay and Le May’s mindlines paradigm. | 5 |
| S6 | **Researcher characteristics and reflexivity** | Paragraph included about research team and reflexive accounting. To address bias arising from personal beliefs about management of suspected maternal sepsis and AMR. | 5 |
| S7 | **Context** | This research involved clinicians from England, Scotland and Wales working in NHS hospitals providing care for pregnant women during labour. | 5 |
| S8 | **Sampling strategy** | Study information was shared via national and local clinical networks. A snowball sample ensued from which a purposeful sampling matrix was used to ensure representation from England, Scotland, and Wales, hospitals with <3,000, 3,000-6,000, >6,000 births per annum, different grades, and length of NHS service. | 5 |
| S9 | **Ethical issues pertaining to human subjects** | This research received Research Ethics Committee approval from the University of Liverpool (Ref:13856). | 5 |
| S10 | **Data collection methods** | 24 semi-structured interviews. | 5 |
| S11 | **Data collection instruments and technologies** | Semi-structured interview guide exploring clinicians’ beliefs, views and experiences of broad-spectrum antibiotic prescribing for suspected maternal sepsis. | 5 |
| S12 | **Units of study** | Clinicians | 5 |
| S13 | **Data processing** | Interviews were recorded and transcribed using MSTeams, verbatim transcripts were checked before uploading to MAXQDA for data management and analysis |  |
| S14 | **Data analysis** | Framework Approach. | 6 |
| S15 | **Techniques to enhance trustworthiness** | Thematic framework was constructed to encapsulate a priori issues (informed by the questions asked), emergent issues (raised by participants) and analytical themes (arising from recurrence or patterning in data) from first 10 interviews. The final framework matrix was agreed by two authors (CK, AM). All authors reviewed the final themes. | 6 |
| **Results/findings** | | |  |
| S16 | **Synthesis and interpretation** | Themes and subthemes are outlined in results section, Figure 2, and Tables 2,3 and 4. | 7-13 |
| S17 | **Links to empirical data** | Quotes integrated within results and Tables 2,3 and 4. |  |
| **Discussion** | | |  |
| S18 | **Integration with prior work, implications, transferability, and contribution(s) to the field** | Discussion section has paragraphs detailing what this study adds and findings in relation to other studies. | 13-14 |
| S19 | **Limitations** | Paragraph in the Discussion section. | 14 |
| **Other** | | |  |
| S20 | **Conflicts of interest** | None declared. | 16 |
| S21 | **Funding** | This work was supported by a UK Health Security Agency award (Ref: PB0938). | 16 |

Source: Brien B, Harris I, Beckman T, Reed D, Cook D. Standards for reporting qualitative research. Acad Med. 2014;89(9):1245-51.
