# Supplementary material for: Clinicians’ decision-making about broad-spectrum antibiotic prescribing for suspected maternal sepsis during childbirth in the UK: a qualitative study
Source: BMJ Open. 2026 Jul 17;16(7):e110559. doi: 10.1136/bmjopen-2025-110559 (PMC13384126; doi:10.1136/bmjopen-2025-110559)
Supplement: online supplemental file 2 [file bmjopen-16-7-s002.pdf]

RESEARCH PROTOCOL

Qualitative study exploring what knowledge informs decisions about broad spectrum antibiotic prescribing for suspected sepsis in women admitted to hospital for childbirth

Preparatory work funded by the UK Health Security Agency
Towards innovative management of maternal sepsis to tackle antimicrobial resistance in maternity care
and development of a Non-Invasive Monitoring Biosensor for Labour and Early Life (NIMBLE-Pregnancy)

Research Reference Numbers

University of Liverpool Research Ethics Committee:	13856
Sponsor:	University of Liverpool
Funder:	UK Health Security Agency
Chief Investigator:	Dr Carol Kingdon

HRA protocol compliance declaration: This protocol has regard for HRA Guidance

Study Flow Chart

Identification of potential participants

- Information shared with pre-existing regional trainee and national consultant groups and on social media.
- Potential participants self-identify to clinical lead, who answers any questions and confirms eligibility.
- With permission, clinical lead passes potential participant's email address on for the lead researcher to contact.

↓

Recruitment and consent of participants

- Lead researcher contacts potential participant via email to arrange a date for interview via MS Teams, with a copy of the participant information sheet and consent form attached for information.
- The scheduled MS TEAMS call will have two parts. Part 1 is the taking of valid and informed consent by the researcher.
- Consent will be audio recorded and electronically documented.

↓

Semi-structured interview

- Part 2 of the MS TEAMS call will be the semi-structured interview.
- Interviews will last approximately 45 minutes.
- Interviews will be audio or video recorded depending on participant preference.

↓

Data Management, Analysis and Dissemination

- All interviews will be scheduled, conducted, and recorded using a University of Liverpool MS TEAMS account for regulatory compliant data management of audio, transcription, and data analysis files.
- Electronic consent forms and consent recordings will be saved on the University of Liverpool managed network in password protected folders.
- Interview recordings and transcription files will be saved on the University of Liverpool Managed Network in a separate password protected folder to consent forms.
- Anonymised transcripts will be uploaded to MAXQDA for analysis on a University of Liverpool password protected computer.
- Preliminary findings will be reported to the funder by the 31st March 2024.

Contents

1. Introduction 4
2. Background 4
3. Rationale 4
4. Theoretical framework 4
5. Research Aim 5
6. Study Design, Methods of Data Collection and Analysis 5
7. Study setting 6
8. Sample and Recruitment 6
9. Ethical and Regulatory Considerations 7
10. End of study and Dissemination Policy 8
11. References 9

1. Introduction

This protocol describes a "Qualitative study exploring what knowledge informs decisions about broad spectrum antibiotic prescribing for suspected sepsis in women admitted to hospital for childbirth" and provides information about study procedures and governance requirements. This study will adhere to the principles outlined in the UK Policy Framework for Health and Social Care Research. It will be conducted in compliance with the protocol, the Data Protection Act 2018 and the UK GDPR as amended from time to time and any successor legislation in the UK and any other directly applicable regulation relating to data protection and privacy as well as any other regulatory requirements as appropriate.

2. Background

Labour is a risky time for infection, especially once a woman's waters are broken as there is a direct pathway for pathogens into the uterus where they can infect both mother and baby. For example, with broken waters for greater than 24 hours, there is a 1% risk of serious fetal infection, with 0.5% prior to that.[1] In 2022-3 there were 547,244 births in England.[2] From existing data we estimate 19,000 (3.5%) are suspected of maternal sepsis, and likely started on broad spectrum antibiotics.[3] Front-line clinicians are aware that few of these women go on to develop maternal sepsis, defined as a life-threatening condition defined as organ dysfunction resulting from infection.[4] This is supported by a UK wide obstetric surveillance study of critical care admissions where, in a 2-year period, 1,120 women had severe sepsis/septic shock.[5] Our recent engagement work with obstetric(8), anaesthetic(3) and infectious diseases(1) consultants found triggers for starting broad spectrum antibiotics in labour are not pregnancy specific and clinicians suspect many women receive antibiotics unnecessarily. However, not giving antibiotics to women with suspected sepsis within the specified timeframe is open to criticism as failure to recognise sepsis, late or inappropriate treatment of sepsis can contribute to preventable maternal morbidity and mortality.[6] Those at highest risk of maternal death from sepsis, as with all maternity conditions, are women from lower socioeconomic backgrounds and black and minority ethnic groups. Current national guidance recommends routine observations (heart rate and temperature) and venous maternal lactate measurement.[7] Since 2017 it has been recommended that a lactate of 2.0mmol/l or higher is a risk factor for sepsis, and this contributes to women being started on sepsis bundles which include broad spectrum antibiotics.[8] This study is part of a wider programme, which includes a systematic review,[9] towards better management of maternal sepsis in maternity care.

3. Rationale

The successful implementation of research evidence to support the Government ambitions to tackle antimicrobial resistance requires a better understanding of clinical decision-making in context. This is especially important in maternity care where coexisting government ambitions to tackle sepsis, stillbirth,[10] maternal and neonatal deaths coalesce in the care of pregnant women and their unborn child(ren).

4. Theoretical framework

This study will draw on Gabbay and Le May's concept of mindlines to explore how different kinds of knowledge, experience, values, and behaviours navigate the linear rationalism of guidelines to the complex wisdom of good practice.[11] Mindlines, offer a way of

acknowledging the role of tacit knowledge, together with clinical guidelines, in every-day decision-making processes across four domains:[12]

- **clinical** (for example, diagnosing, prescribing, explaining, referring)
- **management** (for example, handling resources, personnel and the NHS hierarchy, quality assurance, training staff, using the IT system)
- **public-health** (for example, prevention, health promotion, understanding local needs and national concerns)
- **professional self-management** (for example, keeping up to date, reviewing practice, nurturing networks of trusted colleagues, sustaining personal and disciplinary credibility)

5. Research Aim

To explore medical decision-making about prescribing broad spectrum antibiotics, and the role of national guidelines, alongside more tacit forms of knowledge to identify how, and why, over-prescribing may occur.

5.1 Outcomes
Lived experience evidence of the complexity of clinical decision-making in context to inform future grant applications.

6. Study Design, Methods of Data Collection and Analysis

6.1 Design
This is a qualitative research study using semi-structured interviews only.

6.2 Sampling frame
A maximum sample of 25 participants will be sought. Recruitment will end earlier if data saturation is reached with less participants. A purposive sampling matrix will be used to ensure representation across the UK, size of unit (<3,000, 3,000-6,000, >6,000 births per annum), and grade (consultant, ST1-7, and non-training grade doctors) and length of service.

6.3 Data collection method
Semi-structured interviews will be conducted using MS Teams. A semi-structured interview schedule has been designed specifically for the purposes of the study (Appendix 12.1.6). Open questions explore medical decision-making about prescribing broad spectrum antibiotics, and the role of national guidelines, alongside more tacit forms of knowledge to identify how, and why, over-prescribing may occur. Probes will be used relating to Gabbay and Le May's four domains (clinical, management, public health, professional self-management). Relevant issues emerging from the discussion that are not on the interview schedule may also be explored. Interviews will last approximately 45 minutes. Interviews will be audio or video recorded depending on participant preference.

6.4 Data analysis
Interviews will be recorded, transcribed, and uploaded into MAXQDA for thematic qualitative data analysis using the Framework Approach.[13] Data collection and interpretation will be simultaneous. The Framework Approach to thematic analysis involves the following key stages.[13-14]

1. **Familiarization** to gain an overview of the richness, breath, depth and diversity of data and begin the process of abstraction and conceptualization;
2. **Identifying a thematic framework** encapsulating a priori issues (informed by the questions asked), emergent issues (raised by participants) and analytical themes (arising from recurrence or patterning in data);
3. **Indexing** of the data using the framework to categorise data into manageable 'bites' (codes in MAXQDA);
4. **Charting** of indexed/coded data from MAXQDA, according to a priori research questions and how best to present findings to inform further research into analytical themes;
5. **Mapping and interpretation**, the crux of the analysis, whereby we will weigh up the salience and dynamics of issues, searching for overall structure via defining concepts, mapping the range and nature of phenomenon, creating typologies, finding associations, providing explanations, and developing strategies.

CK will lead data analysis, with input from AM at all stages. The views of the wider study management committee will be sought at stage 2 and 5.

6.5 Data management

All research data will be stored within University of Liverpool data storage infrastructure and will comply with the research data management policy [researchdatamanagementpolicy.pdf (liverpool.ac.uk)](researchdatamanagementpolicy.pdf) All interviews will be scheduled, conducted, and recorded using a University of Liverpool MS TEAMS account for regulatory compliant data management of audio, video and transcription files. Electronic consent forms and consent recordings will be saved on the University of Liverpool managed network in password protected folders. Interview recordings and transcription files will be saved on the University of Liverpool Managed Network in a separate password protected folder to consent forms. Partial-anonymised transcripts will be uploaded to MAXQDA for analysis on a University of Liverpool password protected computer. All data will be password protected and accessible by the necessary members of the research team only. Data which will be used for subsequent analysis will undergo full anonymisation by decoupling of the participant number. On completion of the study, all study data including consent forms and other participant identifiable information will be stored for a minimum of 10 years. The University of Liverpool Active DataStore provides a centralised, secure, supported data storage facility for electronic data, with ongoing access for the life span of a project. This space, and its underlying technical infrastructure are fully supported by IT Services who continually review and improve security arrangements. The Active DataStore has many layers of protection, with data replicated between two secure physical locations and backed up regularly. Additionally, a regular tape backup is made to a third physical location, and segregated from the public network both physically and logically. Data is encrypted in transit using SSL. The research data will be accessed only by named members of the research team granted password protected access to the Active DataStore by the Chief Investigator.

7. Study setting

The study will involve doctors working in the United Kingdom's National Health Service, who volunteer to participate. Potential participants will self-identify. Screening will ensure representation from across the UK.

8. Sample and Recruitment

8.1 Eligibility

- Doctors currently working in a UK NHS intrapartum maternity care setting.
- Volunteer and provide valid and informed consent.

8.2 Screening and recruitment

Snowball sampling will be used. Information will be shared with pre-existing regional trainee and national consultant groups online, via email and via whatsapp (Advert Appendix 12.1.3, Information Sheet Appendix 12.1.4). Potential participants will self-identify to the clinical lead (AM), who will answer any questions, confirm eligibility, and with permission pass on their email address for the lead researcher (CK) to make contact. Alternatively, participants can directly contact the lead researcher (CK).

8.3 Informed consent

The lead researcher will contact potential participants via email to arrange a date for interview via MS Teams, with a copy of the participant information sheet and consent form attached for information. The scheduled MS TEAMS call will have two parts. Part 1 is the taking of valid and informed consent by the researcher. This will be audio recorded and electronically documented.

9. Ethical and Regulatory Considerations

This is a non-interventional study using qualitative research methods (Appendix 12.1.7 HRA Is this research?) This research will involve NHS staff recruited by virtue of their professional role. However, there is no NHS organisation involved as a site and no formal confirmation of NHS capacity and capability is required.

9.1 Ethics Approval

The Health Research Authority (HRA) Decision Tool indicated that this study does not require NHS Research Ethics Committee (REC) review in England, Scotland, Wales, or Northern Ireland (Appendix 12.1.8, 12.1.9, 12.1.10., 12.1,11). Before the start of the study, a favourable opinion will be sought from the University of Liverpool REC for the study protocol, informed consent form and other relevant documents (all documents listed in listed in appendix 12.1).

9.2 Health Research Authority Approval

We sought the advice of the HRA and the Head of Research, Development and Innovation at Liverpool Women's NHS Foundation Trust. Appendix 12.1.12 confirms HRA approval is not required.

9.3 Data Protection and Participant Confidentiality

Under UK data protection legislation, the University acts as the Data Controller for personal data collected as part of the University's research. The lead researcher acts as the Data Processor for this study. The Chief Investigator will preserve the confidentiality of participants taking part in the study and will abide by the Data Protection Act 2018 and the UK GDPR as amended from time to time and any successor legislation in the UK and any other directly applicable regulation relating to data protection and privacy. In addition to compliance with the Data Protection Act and the GDPR, privacy will be respected in accordance with the Human Rights Act 1998 and in common law in relation to confidential personal information. The only place participant identifiable data will be held is in the study recruitment log (minimal contact details) and on the electronic consent forms and audio recording. These will be password protected and only staff directly related to the study will access the file (CK, AM). Minimal demographic information will be collected next to unique study ID number (not name) on the study database. We do not plan to transmit the data outside the university, as all staff working with the data will have University of Liverpool substantial or honorary contracts. The data will be archived for a minimum of 10 years.

9.4 Indemnity and Intellectual Property

This study is limited to recruiting NHS staff as participants and does not require NHS REC review, meaning proof of insurance/indemnity for the management of a study is not required by the HRA. The University of Liverpool holds Indemnity and insurance cover with Newline Insurance Company. A collaboration agreement will be put in place, so foreground IP for the NIMBLE Pregnancy device is owned by the University of Liverpool, including an agreement to cross license and fair revenue share with Imperial College London.

9.5 Risk Assessment

The University of Liverpool's Basic Risk Assessment Form for Projects has been completed (Appendix 12.1.14). This study involves minimal risk. Participants are NHS staff volunteers. As part of the recruitment process, we will take valid and informed consent. We do not require access records to collect personal or sensitive confidential information. There are no intrusive interventions. The interviews pose minimal risk to researcher safety with interviews conducted on-line via MS Teams, and minimal risk of duty to disclose as questions relate to discussing their area of expertise, national guidance, and general thought processes, rather than specifics of patient care. Plans to minimise risk include:

- Consideration of the relationship between a potential participant and the "recruiter" to ensure this process is free from undue influence.
- There will be no coercion or unacceptable inducement.
- Only very limited personal data necessarily for arranging interviews will be collected.

All potential participants will receive an information sheet that provides details as to what participation involves, the voluntary nature of participation, confidentiality, anonymity, and withdrawal - participants will have the option to withdraw their data up to seven days post interview. The information sheet and consent form will be reviewed at the start of the interview and then, if the participant is still willing to proceed, it will be signed by the researcher on the participant's behalf. The consent procedure will be audio recorded (including participant's responses and agreement) for verification purposes. The researcher will ask the participant the consent statements, noting the answers of the participant on a consent form which has a final statement "I have accurately read out the information sheet to the potential participant and, to the best of my ability, ensured that the participant understands to what they are freely consenting", then this will be signed and dated by the researcher. The consent form includes a section related to data share. No controller/processor relationship will be required between the University of Liverpool and an NHS organisation, and GDPR does not require a legally binding agreement.

10. End of study and Dissemination Policy

The end of the study will be the 31st March 2024.

10.1 Outputs

Data from this study will be owned by the University of Liverpool. Initial findings will be available by 31st March 2024. A full report of the study will be produced and submitted for peer-reviewed publication and shared at national and international conferences of interest to obstetricians, infectious disease and anaesthetic decision makers. The funders will be acknowledged on any publication. Each participant will be offered the opportunity to receive a copy of the publication of the results of the study.

10.2 Authorship eligibility guidelines and any intended use of professional writers

The authors of this protocol will be eligible for authorship for any publications arising from this study. We will follow The International Committee of Medical Journal Editors recommendations.

10.3 Archiving

Data and all appropriate documentation will be stored for a minimum of 10 years after the completion of the study.

11. References

1. National Institute for health and Care Excellence. Preterm labour and birth. NICE guideline Nice Guideline 25. NICE, 2015. https://www.nice.org.uk/guidance/ng25
2. NHS England. NHS Maternity Statistics, England, 2022-23 - NHS Digital
3. Abutheraa, N., Grant, J. & Mullen, A.B. Sepsis scoring systems and use of the Sepsis six care bundle in maternity hospitals. BMC Pregnancy Childbirth 21, 524 (2021).
4. World Health Organization Statement on maternal sepsis (who.int)
5. Acosta CD, Harrison DA, Rowan K, et al. Maternal morbidity and mortality from severe sepsis: a national cohort study. BMJ Open 2016;6:e012323.
6. Knight M, Bunch K, Felker A, Patel R, Kotnis R, Kenyon S, Kurinczuk JJ (Eds.) on behalf of MBRRACE-UK. Saving Lives, Improving Mothers' Care Core Report - Lessons learned to inform maternity care from the UK and Ireland Confidential Enquiries into Maternal Deaths and Morbidity 2019-21. Oxford: National Perinatal Epidemiology Unit, University of Oxford 2023
7. Royal College of Obstetricians and Gynaecologists. Sepsis in Pregnancy, Bacterial (Green-top Guideline No. 64a) April 2012. Available from: https://www.rcog.org.uk/media/ea1p1r4h/gtg_64a.pdf
8. National Institute for health and Care Excellence. Sepsis: recognition, diagnosis and early management. Nice Guideline 51. NICE, 2016. 2017 Update. Available from: https://www.nice.org.uk/guidance/NG51
9. Benjamin Greenfield, Carol Kingdon, Richard Jackson, Yamikani Chimwaza, David Freeman, Damien Ming, David Lissauer, Alison Holmes, Abi Merriel. Maternal lactate during pregnancy, labour, and the immediate postpartum period: a systematic review and meta-analysis. PROSPERO 2023 CRD42023494748
10. Department of Health and Social Care, Safer Maternity Care. Progress Report 2021, June 2021, p18-19 Available from: https://www.england.nhs.uk/wp-content/uploads/2021/03/agenda-item-9.4-safer-maternity-care-progress-report-2021-amended.pdf
11. Gabbay J, le May A. Practice-based evidence for health care: clinical mindlines. Abingdon: Routledge; 2011.
12. John Gabbay, Andrée le May. Mindlines: making sense of evidence in practice. British Journal of General Practice 2016; 66 (649): 402-403.
13. Ritchie J., Spencer L. (1994). Qualitative data analysis for applied policy research. In Bryman A., Burgess R. G. [Eds.], *Analysing qualitative data* (pp. 173–194). London: Routledge.Ritchie, J. & Spencer, L. 1994. Qualitative data analysis for applied policy research" by Jane Ritchie and Liz Spencer in A.Bryman and R. G. Burgess [eds.] "Analyzing qualitative data", 1994, pp.173-194.
14. Gale N. K., Heath G., Cameron E., Rashid S., Redwood S. (2013). Using the Framework method for the analysis of qualitative data in multi-disciplinary health research. *BMC Medical Research Methodology*, 13(1), 117.
