# Supplementary material for: Clinicians’ decision-making about broad-spectrum antibiotic prescribing for suspected maternal sepsis during childbirth in the UK: a qualitative study
Source: BMJ Open. 2026 Jul 17;16(7):e110559. doi: 10.1136/bmjopen-2025-110559 (PMC13384126; doi:10.1136/bmjopen-2025-110559)
Supplement: online supplemental file 3 [file bmjopen-16-7-s003.pdf]

Supplementary File 3: Semi-structured interview guide

Semi-Structured Interview Schedule

Qualitative study exploring what knowledge informs decisions about broad spectrum antibiotic prescribing for suspected sepsis in women admitted to hospital for childbirth

Version number & date: Version 1.0 04/01/24
Research ethics approval number: 13856
Name of researchers: Dr Carol Kingdon, Dr Abi Merriel, Professor Alison Holmes

All interviews will be scheduled and conducted from University of Liverpool's Microsoft Teams.

Introduction

Researcher states their name, research experience, and role in the study, before reiterating key information from the Participant Information sheet and consent form (shared in advance):

- Aim of the study
- Interviewee free to refuse to answer a question and can end the interview at any point
- Interviewee able to withdraw their data up to seven days post interview
- Non-identifying data will be shared in reports of study findings
- Personal information is known only to the research team
- Interviews will be audio recorded with camera's on or off depending on interviewee's preference
- Valid and informed consent will be recorded with video cameras off

Consent

Researcher shares a copy of the consent form and seeks agreement to electronically acknowledge/sign the consent form on their behalf.

Interview questions

1. In your view, what are the pros and cons of broad antibiotic prescribing for suspected maternal sepsis during labour and childbirth?
   - For the mother-baby dyad
   - For management (practicalities)
   - For public health (local needs, national concerns)
   - For professional self-management

2. In your view, what are the most important factors informing doctors' decision-making process to prescribe broad-spectrum antibiotics for suspected sepsis in women admitted to hospital for childbirth?

- What kinds of knowledge do you draw on in your decision-making process to prescribe?
- What kinds of knowledge do you draw on in your decision-making process <u>not</u> to prescribe?

3. In your view, how have doctors decision-making processes regarding broad spectrum antibiotic prescribing changed over time?-
 - Which kinds of knowledge can influence a shift in your decision-making process to prescribe broad spectrum antibiotics?
 - Which kinds of knowledge can influence a shift in your decision-making process not to prescribe broad spectrum antibiotics?

4. In the current context, what action(s) would you suggest to address over-prescribing of broad spectrum antibiotics for suspected sepsis in women admitted to hospital for childbirth?

5. Some questions about you:
 - Which UK nation do you work in? England, Scotland, Wales, Northern Ireland.
 - How many years have you worked in intrapartum maternity care in the NHS?

Specialist Trainees	Consultants	Non-training grade Drs
0-4	0-4	0-4
5-9	5-9	5-9
10+	10-19	10-19
	20-29	20-29
	30+	30+

 - What is the size of unit you currently work in? 3,000, 3,000-6,000, >6,000 births per annum

6. Is there anything else you would like to tell us about your views or experiences of
 - medical decision-making about prescribing broad spectrum antibiotics
 - the role of national guidelines
 - other forms of knowledge
 - how or why, over-prescribing may occur

Re-confirm interviewee would, or would not like to receive a copy of the results.

Thank you.
