# Supplementary material for: Clinicians’ decision-making about broad-spectrum antibiotic prescribing for suspected maternal sepsis during childbirth in the UK: a qualitative study
Source: BMJ Open. 2026 Jul 17;16(7):e110559. doi: 10.1136/bmjopen-2025-110559 (PMC13384126; doi:10.1136/bmjopen-2025-110559)
Supplement: Supplementary file 4 [file bmjopen-2025-110559.reviewer_comments.pdf]

PEER REVIEW HISTORY

BMJ Open publishes all reviews undertaken for accepted manuscripts. Reviewers are asked to complete a checklist review form and are provided with free text boxes to elaborate on their assessment. These free text comments are reproduced below.

ARTICLE DETAILS

Title (Provisional)

Clinicians' decision-making about broad-spectrum antibiotic prescribing for suspected maternal sepsis during childbirth in the UK: A qualitative study

Authors

Kingdon, Carol; Greenfield, Benjamin; Cunningham, Caroline; Freeman, David; Ming, Damien; Lissauer, David; Holmes, Alison; Merriel, Abi

---

VERSION 1 - REVIEW

Reviewer 1

Name Remuzzi, Giuseppe

Affiliation Istituto di Ricerche Farmacologiche Mario Negri IRCCS, Centro Anna Maria Astori, Science and Technology Park Kilometro Rosso

Date 05-Dec-2025

COI None

Summary of paper

This is a qualitative study, based on interviews obtained from 24 clinicians, sampled from relevant professions with roles in

decision-making, to describe how they navigate intrapartum guidelines, under uncertain evidence about broad-spectrum antibiotic prescribing for suspected maternal sepsis during childbirth. They were chosen from hospitals across England, Scotland and Wales in March-June 2024. They found that overtreatment may be inevitable until better evidence to support decision-making. This study may be of importance, highlighting an urgent need to optimize early identification and treatment of sepsis and the use of broad-spectrum antibiotics. However, it leaves several important questions unanswered, which should be addressed.

Methods

- A sample of 24 maternity clinicians was selected: 15 obstetricians, 4 anesthetists, 3 midwives and 2 microbiologists. It is unclear how the study size was arrived at and which criteria were used for participants' selection.

- Please, provide a quantitative definition of 'overtreatment'.

- It is unclear whether some of the sampled clinicians refused to participate, being replaced by others who agreed to perform the interview. If yes, could the Authors provide information on this point?

- The Authors failed to specify whether the interviews were anonymous or not. Of concern, participants belonging to professions with a very low number of interviews – i.e. the four anesthetists, the three midwives and the two microbiologists – are easily recognizable, even on condition of anonymity. This aspect may represent a critical concern, leading to conscious or unconscious cognitive biases (e.g. complacency bias).

Results

- At times the text is difficult to follow. An effort to summarize the most important study findings, ideally on one or more tables, could be helpful.

- At page 11, line 3, the title of the paragraph refers to the mother-baby dyad as 'two patients': this may be ambiguous. Please, replace 'two patients' with 'mother-baby dyad'.

Discussion

-A major limitation of the study is that several biases are inherent in qualitative research. Personal experiences or preconceived notions of researchers and clinicians who agree to participate can introduce bias, undermining the study's validity. A comment on this important point should be provided.

-The subjective nature of data collection and analysis may lead to generalizability concerns and to challenges in extending findings beyond the specific context of the study. The Authors should clarify the extent to which their study findings are generalizable.

**Reviewer**	**2**
**Name**	**Henley, Josie**
**Affiliation**	**Cardiff University, School of Social Sciences**
**Date**	**12-Mar-2026**
**COI**	**None**

The article makes an important contribution both to maternal/neonatal care and antibiotics stewardship. With some very minor amendments I suggest that it is accepted.

The term 'mindlines' is defined on page 38 lines 15-16, however also appears in the abstract, page 34, line 49 and 'strengths and limitations' section on page 35 line 27. As I was unfamiliar with the term, it was not entirely clear what it referred to, e.g. perhaps a clinical decision tool or software. I would suggest replacing the term in the abstract and what this study adds section with something more easily recognisable, e.g. clinicians' tacit knowledge, until the term is defined.

In the limitations on page 46, line 17, it is noted that there was an absence of women's views in the study. However, Table 1 participant characteristics clearly indicates 17 female participants. I assume therefore that "women" in this context refers to non-clinicians who have used maternity services. Given there is quite an overlap between clinicians and people who have used or might in the future use maternity services, I suggest substituting "women" with "patients / public" or non-clinicians or another term to make this clear.

Some of the quotes from participants appear to not be attributed. E.g. at the beginning of the results section, page 39 lines 31-45, quotes are embedded in the paragraph text. The first quote "There's no actual clear evidence..." is attributed to ID05, however the following paragraph contains several quotes that are attributed to nine, twelve, and three participants. They seem too long and specific to be themes/sub-themes (and do not appear in Table 2), but not quite believable that nine or twelve participants used exactly the same wording to make the point. This should be made more clear, e.g. use the quote and attribute it to the participant, then state that eight other participants said something that would align with that quote. Alternatively, use the method in sub-theme 2.1 page 42, lines 16-19, where quotes are attributed by reference to Table 3.

Some proofreading is required, e.g. both MEOWS and MEOWSS appear on page 42, line 47. I believe that MEOWS is correct, however if MEOWSS is also used (e.g. Score System), then this should be clearly labelled and the same acronym used consistently.

VERSION 1 - AUTHOR RESPONSE

Thank you for your email dated 9th April 2026. Please find our response to the comments below.

Reviewer	Reviewer’s comment	
Reviewer 1:<br>Dr. Giuseppe Remuzzi, Istituto di Ricerche Farmacologiche Mario Negri IRCCS	This is a qualitative study, based on interviews obtained from 24 clinicians, sampled from relevant professions with roles in decision-making, to describe how they navigate intrapartum guidelines, under uncertain evidence about broad-spectrum antibiotic prescribing for suspected maternal sepsis during childbirth. They were chosen from hospitals across England, Scotland and Wales in March-June 2024. They found that overtreatment may be inevitable until better evidence to support decision-making. This study may be of importance, highlighting an urgent need to optimize early identification and treatment of sepsis and the use of broad-spectrum antibiotics. However, it leaves several important questions unanswered, which should be addressed.	We thank the reviewer for their important comments. We have responded to Reviewer 1’s specific remarks on a point-by-point basis and made corresponding changes to the manuscript/main document.

Reviewer 2:<br>Dr. Josie Henley, Cardiff University	The article makes an important contribution both to maternal/neonatal care and antibiotics stewardship. With some very minor amendments, I suggest that it is accepted.	We thank the reviewer for this comment and their suggested amendments. We have actioned all the changes. These are also detailed below point-by-point and tracked in the manuscript/main document.

Response to Reviewer 1

Item	Reviewer comments	Author's response
1	Method	
1.1	A sample of 24 maternity clinicians was selected: 15 obstetricians, 4 anaesthetists, 3 midwives and 2 microbiologists. It is unclear how the study size was arrived at and which criteria were used for participants' selection.	Thank you for this comment and the opportunity to clarify.<br><br>Per protocol, funding award and reviews of optimum sample size for qualitative interview research, we sought a maximum sample size of 25 interview participants. The purposive sampling frame was designed to principally ensure representation across geographical sites, size and grades of obstetricians because they are the main prescribers of broad-spectrum antibiotics during labour in the UK. The inclusion of anaesthetists and microbiologists was proportionate to practice. We submitted an amendment to include midwives based on the emerging findings from the first 15 interviews. We applied the same principle to sampling across professions (geographical spread and representation of different grades). Thus, our final sample includes 15 obstetricians (main prescribers in practice, representation across place and grades), 4 anaesthetists (representation across place and grades), 3 midwives (from North, South and Central England, with representation across grades) and 2 microbiologists (from North and South of England, both consultant grade as per practice patterns).<br>Several recent systematic reviews have examined samples size in qualitative research. We drew on their findings when writing our protocol. The findings suggest the following sample size guidelines: theme saturation (9 interviews; 4 focus groups), meaning saturation (24 interviews; 8 focus groups), theoretical saturation (20–30+ interviews), meta theme saturation (20–40 interviews per site), and saturation in salience (10 exhaustive free lists). We sought theme and meaning saturation. To address this comment, we have:<br><br>1. Added three sentences to the manuscript in the sample and setting section.<br><br>- Several recent systematic reviews have examined sample size in qualitative research, which we drew on when writing our protocol. Per protocol (Supplementary File 3), funding award and reviews of optimum sample size for qualitative interview research, we sought a maximum sample size of 25 interview participants.45-48<br>- The purposive sampling frame was principally designed to ensure breadth and depth of representation amongst obstetricians because they are the main prescribers of broad-spectrum antibiotics during labour in the UK. The inclusion of anaesthetists, midwives and microbiologists applied the same principles of geographical spread.<br><br>2. Moved two existing references in the manuscript and added Hennink M, Kaiser BN. Sample sizes for saturation in qualitative research: A systematic review of empirical tests. Soc Sci Med. 2022 Jan;292:114523. doi: 10.1016/j.socscimed.2021.114523.<br><br>3. Uploaded the protocol as a supplementary file.
1.2	Please, provide a quantitative definition of 'overtreatment'.	We thank the reviewer for this comment. How to define overtreatment is something we have discussed at length during this study. We are aware of 25 years of debate about overdiagnosis, overtreatment and low-value care in medical journals, including the BMJ[1-2] , and the BMJ Quality and Safety[3] and the BMJ's Too much medicine initiative.[4]<br><br>To address this comment, we have added two new sentences to the introduction at the beginning of the second paragraph and a new reference (2 below; 17 in the manuscript). The two new sentences are highlighted in the paragraph below. Because this is where we provide quantitative study findings reporting overtreatment. The paragraph now reads:

		Overdiagnosis and overtreatment are widely acknowledged to be complex problems that are not easy to define. Over-diagnosis and the over-treatment that follows refer to situations where a diagnosis is technically correct under current clinical standards yet is unlikely to benefit the patient. In such cases, the condition identified may never have caused symptoms or harm, and the interventions that follow can expose the patient to unnecessary risks, side effects, or psychological burden.[17] There is evidence of over-treatment of suspected sepsis using broad-spectrum antibiotics across medical specialities where clinicians often make antibiotic decisions whilst facing diagnostic uncertainty.[16-18] General adult inpatient studies report that up to 50% of patients treated with broad-spectrum antibiotics for sepsis do not have sepsis.[16] Up to 70% of Intensive Care Unit (ICU) patients receive antimicrobial therapy, despite up to half never having infection confirmed.[18] In maternity, a 2020 Scottish cohort study reported a sepsis incidence of 331 per 10,000 pregnancies,[19] far higher than previous studies reporting 10 per 10,000 pregnancies.[19-20] The study included all suspected or confirmed cases treated with antibiotics. In our recent study of 22,662 births, 36 of 839 women treated for suspected sepsis during labour had pathogenic positive blood cultures.[21] During labour, physiological changes can make commonly used biomarkers difficult to interpret. National guidelines in the UK include modifications for pregnant women but rely on observations from non-pregnant populations.[22-25] As an example, NICE recommend Lactate ≥2.0 mmol/l as a diagnostic marker. However, during the exertion of labour and birth, it is likely that a mother's lactate will rise.[26-27] The 2024 RCOG Guideline recognises this and mentions ≥4.0 mmol/l.[24] In 2024, NICE also revised its guideline, updating its recommendations for stratifying risk to optimise appropriate antibiotic administration and address overuse, but recommendations remain unchanged for pregnant populations because of insufficient evidence.[28]<br><br>1. Carter SM, Rogers W, Heath I, et al. The challenge of overdiagnosis begins with its definition. BMJ 2015;350:h869.<br><br>2. Pathirana T, Clark J, Moynihan R. Mapping the drivers of overdiagnosis to potential solutions. BMJ 2017;358:j3879<br><br>3./17. Armstrong N. Overdiagnosis and overtreatment as a quality problem: insights from healthcare improvement research. BMJ Qual Saf. 2018 Jul;27(7):571-575. doi: 10.1136/bmjqs-2017-007571. Epub 2018 Mar 23.<br><br>4. Too much medicine | The BMJ
1.3	It is unclear whether some of the sampled clinicians refused to participate, being replaced by others who agreed to perform the interview. If yes, could the Authors provide information on this point?	Thank you for the opportunity to clarify, Snowball sampling was used. Information was shared with regional trainee and national consultant groups online, via email and whatsapp. Potential participants self-identified to the clinical lead (AM), who answered any questions, confirmed eligibility, and passed on their email address for the lead researcher (CK) to make contact. Obstetric participants shared study information with colleagues from other professional groups. To address this comment we have added to the existing sentence Study information was shared via national clinical networks, and participants self identified to the study team encapsulates this, so we have not added further detail to the manuscript. We are happy to do so if the reviewer or editor feels is essential.
1.4	The Authors failed to specify whether the interviews were anonymous or not. Of concern, participants belonging to professions with a very low number of interviews – i.e. the four anesthetists, the three midwives and the two microbiologists – are easily recognizable, even on condition of anonymity. This aspect may represent a critical concern, leading to conscious or unconscious cognitive biases (e.g. complacency bias).	Thank you for this comment. All interviews were conducted by the lead researcher (CK), a non-clinician after gaining informed consent. All potential participants received an information sheet stating: Interview transcripts will be semi-anonymized for data analysis. Every effort will be made to fully anonymise quotes when reporting overall findings, prior to giving their consent to participate.<br><br>To mitigate conscious or unconscious cognitive bias across the team during the analysis process:<br>1. All interview transcripts were anonymised by CK prior to analysis<br>2. The initial framework matrix was agreed upon by two authors.<br>3. All authors reviewed the final themes.<br>This process is documented in the data analysis section of the manuscript. Please also see our response to comment 3.1 and use of reflexivity.<br><br>Every effort has been made to ensure anonymity in the data submitted for publication.
2.0	Results	
2.1	At times the text is difficult to follow. An effort to summarize the most important study findings, ideally on one or more tables, could be helpful.	We have added a Key points Box (Figure 3) in response to this comment. We have also carefully proofread the manuscript and made some typographical changes in response to this comment and Reviewer 2's comment 4.

		**Figure 3: Key Points**<br><br>**What is already known on this topic**<br>1. Maternal deaths from sepsis are rare in the UK but remain a persistent leading cause of preventable death in MBRRACE-UK (Mothers and Babies: Reducing Risk through Audits and Confidential Enquiries across the UK).<br>.<br>2. Physiological changes in pregnant women mean that commonly used markers for sepsis can be difficult to interpret during labour, while research in other areas of medicine has shown diagnostic uncertainty can influence broad-spectrum antibiotic prescribing impacting antimicrobial stewardship efforts and potentially contributing to antimicrobial resistance (AMR).<br><br>3. The UK Government is far from achieving its containment vision for AMR, with uncertainty about the extent of healthcare workers' knowledge or the contextual factors that determine the effectiveness of decision support interventions for antibiotic prescribing.<br><br>**What this study adds**<br>1. Case study of how maternity clinicians navigate intrapartum guidelines in the context of uncertain evidence, demonstrating the depth of reasons for prescribing broad-spectrum antibiotics during labour, and the power of sepsis guidelines.<br><br>2. Qualitative data from a national sample of obstetricians, midwives, microbiologists and obstetric anaesthetists that shows why over-treatment of suspected maternal sepsis during labour is inevitable and how AMR decision support interventions will need to become embedded in clinical guidelines and clinicians' mindlines.<br><br>3. Evidence of an unmet need to include pregnant women in general infection studies and an urgent need to develop pregnancy-specific evidence base for labour towards point-of-care diagnostic tests for decision support to optimise early identification and treatment of sepsis and the use of broad-spectrum antibiotics.
2.2	At page 11, line 3, the title of the paragraph refers to the mother-baby dyad as 'two patients': this may be ambiguous. Please, replace 'two patients' with 'mother-baby dyad'.	Thank you for this suggestion. We have changed this in:<br><br>1. Page 11, line 3 as suggested<br>2. Figure 2<br>3. Table 3<br>4. Discussion
3.0	Discussion	
3.1	A major limitation of the study is that several biases are inherent in qualitative research. Personal experiences or preconceived notions of researchers and clinicians who agree to participate can introduce bias, undermining the study's validity. A comment on this important point should be provided.	Thank you for raising this important point. When conducting qualitative research, individuals and teams should engage in reflexivity to account for how subjectivity shapes their inquiry. Reflexivity is an integral part of ensuring qualitative rigour, transparency, and ethical robustness. To make our reflexive accounting clearer:<br><br>1. We have added the sub-heading Reflexivity to the existing paragraph to make it more explicit.<br>2. We have added sentences at the beginning and end of that paragraph, so it now reads:<br><br>When conducting qualitative research, teams should engage in reflexivity to account for how subjectivity shapes their inquiry.[43-44] We are an interdisciplinary team from sociology (CK), obstetrics (AM, BG, DL), infectious diseases (DM, AH), midwifery (CC), and electrochemistry (DF). Our preexisting beliefs encompassed clinical concerns about over-treatment (AM,BG), gaps in knowledge about physiological labour and sepsis (AM,BG,DL,DM,CK), persistent inequalities in maternal deaths from sepsis (CK,AM,CC,DL) and scope for new point-of-care diagnostic technologies during labour to address early sepsis management and AMR (AH,DF,DM,AM,BG,CK).[34] We remained aware of these prior beliefs and took steps to mitigate professional biases. All interviews were conducted by the non-clinician in the team and we chose a rigorous, stepwise approach to data analysis to ensure that we were not

		over-interpreting data that supported prior views, or overlooking data that ran contrary to our beliefs.<br><br>3. Added two additional references<br>43. Olmos-Vega, F. M., Stalmeijer, R. E., Varpio, L., & Kahlke, R. (2023). A practical guide to reflexivity in qualitative research: AMEE Guide No. 149. Medical Teacher, 45(3), 241–251. https://doi.org/10.1080/0142159X.2022.2057287<br>44. Kingdon, C. (2005) Reflexivity: Not just a qualitative methodological research tool. BJM; 13 (10). https://doi.org/10.12968/bjom.2005.13.10.19835
3.2	The subjective nature of data collection and analysis may lead to generalizability concerns and to challenges in extending findings beyond the specific context of the study. The Authors should clarify the extent to which their study findings are generalizable.	This is also an important point, that we welcome the opportunity to clarify in the manuscript. While generalizability is, without a doubt, pertinent to reviews of multiple qualitative studies,[1-2] opinion is divided on whether generalizability is an expected attribute of qualitative research findings in individual studies. Transferability is the more accepted quality criterion for studies such as ours. Transferability has similarities and differences to the quantitative concept of universal generalization.[2-4] Our change to the manuscript therefore reads as follows:<br><br>The findings of this study are not intended to be generalisable. However, the qualitative data we report may be transferable,[57] resonating with clinician's views and experiences beyond the context in which this study was conducted.<br><br>Reference added to manuscript:<br>57. Lincoln, Y. S., & Guba, E. G. (1985). Naturalistic inquiry. Sage.<br><br>References in response:<br>1. Lewin S, Booth A, Glenton C, Munthe-Kaas H, Rashidian A, Wainwright M, Bohren MA, Tunçalp Ö, Colvin CJ, Garside R, Carlsen B, Langlois EV, Noyes J. Applying GRADE-CERQual to qualitative evidence synthesis findings: introduction to the series. Implement Sci. 2018 Jan 25;13(Suppl 1):2. doi: 10.1186/s13012-017-0688-3.<br>2. Leung L. Validity, reliability, and generalizability in qualitative research. J Family Med Prim Care. 2015 Jul-Sep;4(3):324-7. doi: 10.4103/2249-4863.161306.<br>3.Korstjens I, Moser A. Series: Practical guidance to qualitative research. Part 4: Trustworthiness and publishing. Eur J Gen Pract. 2018 Dec;24(1):120-124. doi: 10.1080/13814788.2017.1375092.<br>4. Drisko JW. Transferability and Generalization in Qualitative Research. Research on Social Work PracticeVolume 35, Issue 1, January 2025, 102-110. doi.org/10.1177/10497315241256560
**Response to Reviewer 2**		
**Item**	**Reviewer comments**	**Author's response**
1.	The term 'mindlines' is defined on page 38 lines 15-16, however also appears in the abstract, page 34, line 49 and 'strengths and limitations' section on page 35 line 27. As I was unfamiliar with the term, it was not entirely clear what it referred to, e.g. perhaps a clinical decision tool or software. I would suggest replacing the term in the abstract and what this study adds section with something more easily recognisable, e.g. clinicians' tacit knowledge, until the term is defined.	Thank you for this comment and suggestion which we have changed in the results section of the abstract, and the strengths and limitations sections of the manuscript.
2	In the limitations on page 46, line 17, it is noted that there was an absence of women's views in the study. However, Table 1 participant characteristics clearly indicates 17 female participants. I assume therefore that "women" in this context refers to non-clinicians who have used maternity services. Given there is quite an overlap between clinicians and people who have used or might in the future use maternity services, I suggest substituting "women" with	We thank the reviewer for this observation and our omission. We have changed the manuscript as suggested. It now reads:<br><br>Weaknesses include only three midwife participants (from North, South and Midlands), and the absence of patient and public women's views, although beyond the scope of this study.

	"patients / public" or non-clinicians or another term to make this clear.	
3	Some of the quotes from participants appear to not be attributed. E.g. at the beginning of the results section, page 39 lines 31-45, quotes are embedded in the paragraph text. The first quote "There's no actual clear evidence..." is attributed to ID05, however the following paragraph contains several quotes that are attributed to nine, twelve, and three participants. They seem too long and specific to be themes/sub-themes (and do not appear in Table 2), but not quite believable that nine or twelve participants used exactly the same wording to make the point. This should be made more clear, e.g. use the quote and attribute it to the participant, then state that eight other participants said something that would align with that quote. Alternatively, use the method in sub-theme 2.1 page 42, lines 16-19, where quotes are attributed by reference to Table 3.	Thank you for this comment. The reviewer is right the participants did not use the same wording. We have amended the manuscript to attribute direct quotes. It now reads:<br><br>Nine participants explicitly said that they thought over-prescribing leading to over-treatment occurs in maternity and perceived this as a problem (IDs 02,03,04,05,16,18,20,22,24). Direct quotes aligned to this include “100% I think we overprescribe in maternity” (ID05) and “we are probably over treating quite a lot of people” (ID20). Twelve participants (IDs 06,07,09,10,11,12,13,14,15,17,19,21) were sceptical about current use, but their views were more aligned to this quote “because the risk of not doing it is so high that the benefit-risk equation always favours giving it” (ID09). The views of the other three participants (IDs 01,08,23) were more guarded, reporting they did not recall “it was inappropriate seeing someone that had been started on broad-spectrum antibiotics” (ID01).<br><br>Many participants acknowledged a solution to over-treatment is, as this participant said, “an intervention, bedside test or quick lab result that was binary, sepsis, not sepsis.” (ID20).
4.	Some proofreading is required, e.g. both MEOWS and MEOWSS appear on page 42, line 47. I believe that MEOWS is correct, however if MEOWSS is also used (e.g. Score System), then this should be clearly labelled and the same acronym used consistently.	Our apologies for this typographical error which has been corrected in the manuscript. We have carefully proof-read the tracked and clean manuscripts before uploading.
